# Antimicrobial susceptibility among Gram-positive and Gram-negative organisms collected from the Latin American region between 2004 and 2015 as part of the Tigecycline Evaluation and Surveillance Trial

**DOI:** 10.1186/s12941-017-0222-0

**Published:** 2017-07-12

**Authors:** Silvio Vega, Michael J. Dowzicky

**Affiliations:** 1 Complejo Hospitalario Metropolitano, Caja del Seguro Social, Panama City, Panama; 2 Pfizer Inc, Collegeville, PA 19426 USA

**Keywords:** Gram-negative, Gram-positive, Latin America, Resistance, Surveillance, Susceptibility, Tigecycline

## Abstract

**Background:**

The in vitro activity of tigecycline and comparator agents was evaluated against Gram-positive and Gram-negative isolates collected in Latin American centers between 2004 and 2015 as part of the Tigecycline Evaluation and Surveillance Trial (T.E.S.T.) global surveillance study.

**Methods:**

Minimum inhibitory concentrations (MICs) were determined using the broth microdilution methodology according to the Clinical and Laboratory Standards Institute (CLSI) guidelines. Antimicrobial susceptibility was determined using CLSI breakpoints, except for tigecycline for which the US Food and Drugs Administration breakpoints were used.

**Results:**

A total of 48.3% (2202/4563) of *Staphylococcus aureus* isolates were methicillin-resistant *S. aureus* (MRSA). All MRSA isolates were susceptible to linezolid and vancomycin, and 99.9% (2199/2202) were susceptible to tigecycline. Among *Streptococcus pneumoniae* isolates, 13.8% (198/1436) were penicillin-resistant; all were susceptible to linezolid and vancomycin, and 98.0% (194/198) were susceptible to tigecycline. Susceptibility was >99.0% for linezolid and tigecycline against *Enterococcus faecium and Enterococcus faecalis* isolates. A total of 40.8% (235/576) *E. faecium* and 1.6% (33/2004) *E. faecalis* isolates were vancomycin-resistant. Among the Enterobacteriaceae, 36.3% (1465/4032) of *Klebsiella pneumoniae* isolates, 16.4% (67/409) of *Klebsiella oxytoca* isolates and 25.4% (1246/4912) of *Escherichia coli* isolates were extended-spectrum β-lactamase (ESBL) producers. Of the ESBL-producing *K. pneumoniae* and *E. coli* isolates, susceptibility was highest to tigecycline [93.4% (1369/1465) and 99.8% (1244/1246), respectively] and meropenem [86.9% (1103/1270) and 97.0% (1070/1103), respectively]. A total of 26.7% (966/3613) of *Pseudomonas aeruginosa* isolates were multidrug-resistant (MDR). Among all *P. aeruginosa* isolates, susceptibility was highest to amikacin [72.8% (2632/3613)]. A total of 70.3% (1654/2354) of *Acinetobacter baumannii* isolates were MDR, and susceptibility was highest to minocycline [88.3% (2079/2354) for all isolates, 86.2% (1426/1654) for MDR isolates]. Tigecycline had the lowest MIC_90_ (2 mg/L) among *A. baumannii* isolates, including MDR isolates.

**Conclusions:**

This study of isolates from Latin America shows that linezolid, vancomycin and tigecycline continue to be active in vitro against important Gram-positive organisms such as MRSA, and that susceptibility rates to meropenem and tigecycline against members of the Enterobacteriaceae, including ESBL-producers, were high. However, we report that Latin America has high rates of MRSA, MDR *A. baumannii* and ESBL-producing Enterobacteriaceae which require continued monitoring.

**Electronic supplementary material:**

The online version of this article (doi:10.1186/s12941-017-0222-0) contains supplementary material, which is available to authorized users.

## Background

Resistance among clinically important organisms to antimicrobial agents is severely threatening the repertoire of treatment options for common infections. The challenge is intensified by the fact that several of these organisms are resistant to multiple antimicrobials. Antimicrobial resistance is a global problem, with some regions noted to have higher rates of resistance than others. For example, Latin America is reported to have high rates of extended-spectrum β-lactamase (ESBL) producing Enterobacteriaceae, methicillin-resistant *Staphylococcus aureus* (MRSA), and multidrug-resistant (MDR) *Acinetobacter* spp. [1–4]. Also of concern are carbapenemase-producing *Klebsiella pneumoniae.* There have been many outbreaks in the Latin American region [5], particularly in Panama where there was an outbreak from 2011 to 2013 that was difficult to control [6]. Carbapenemases of the metallo-β-lactamases type, such as NDM-1 and VIM, have also emerged in the region [5, 7]. The lack of effective antibiotics against these multi-resistant strains has resulted in an increased use of colistin, and colistin-resistant strains of Enterobacteriaceae, *Pseudomonas* spp. and *Acinetobacter* spp. are beginning to appear [8].

The Tigecycline Evaluation and Surveillance Trial (T.E.S.T.) is an ongoing global surveillance study that has monitored the in vitro activity of tigecycline and comparator agents since 2004. Tigecycline is a broad-spectrum glycylcycline with activity against Gram-positive and Gram-negative organisms. In this report we examine the activity of tigecycline against Gram-positive and Gram-negative organisms collected from centers across Latin America between 2004 and 2015. Data from isolates collected in Latin America in the earlier years of the T.E.S.T. study have previously been presented. Rossi et al. [9] reported antimicrobial resistance between 2004 and 2007, Fernández-Canigia et al. [10] presented antimicrobial susceptibility between 2004 and 2010 (Gram-negative isolates only), and Garza-González et al. [11] presented susceptibility data for *S. aureus* isolates collected between 2004 and 2010.

## Methods

The Latin American countries that participated in T.E.S.T. were Argentina, Brazil, Chile, Colombia, El Salvador, Guatemala, Honduras, Jamaica, Mexico, Nicaragua, Panama, Puerto Rico and Venezuela. Not all study centers submitted isolates during all study years. All body sites were acceptable sources for isolate collection and a maximum of 25% of isolates could be from urine. Isolates were collected from both inpatients and outpatients with documented hospital- or community-acquired infections, and one isolate was permitted per patient.

Detailed materials and methods for the T.E.S.T. study have been described in previous publications (e.g. [12]). Isolate identification and susceptibility testing were performed at the individual centers. Minimum inhibitory concentrations (MICs) were determined using the broth microdilution methodology according to the Clinical and Laboratory Standards Institute (CLSI) guidelines [13]. Antimicrobial susceptibility was determined using breakpoints approved by the CLSI [14], except for tigecycline for which the US Food and Drugs Administration (FDA) breakpoints were used [15]. When determining *Streptococcus pneumoniae* susceptibility to penicillin, oral penicillin V breakpoints were used. In 2006, four antimicrobials (azithromycin, clarithromycin, erythromycin and clindamycin) were added to the *S. pneumoniae* T.E.S.T. panel and, where available, isolates were tested retrospectively.

ESBL production among *Klebsiella* spp. and *Escherichia coli* were determined by IHMA according to CLSI guidelines using cefotaxime, cefotaxime–clavulanic acid, ceftazidime and ceftazidime–clavulanic acid disks. *Haemophilus influenzae* isolates were tested for β-lactamase production using center specific methodology.

In this study, MDR was defined as resistance to three or more classes of antimicrobial agents. The classes used to define MDR *Acinetobacter baumannii* were aminoglycosides (amikacin), β-lactams (cefepime, ceftazidime, ceftriaxone or piperacillin–tazobactam), carbapenems (imipenem or meropenem), fluoroquinolones (levofloxacin) and tetracyclines (minocycline). The classes used to define MDR *Pseudomonas aeruginosa* were aminoglycosides (amikacin), β-lactams (cefepime, ceftazidime or piperacillin–tazobactam), carbapenems (imipenem or meropenem) and fluoroquinolones (levofloxacin).

## Results

Data are presented for a total of 31,933 isolates collected in Latin America between 2004 and 2015 (Table 1); 9918 were Gram-positive and 22,015 were Gram-negative. The majority of isolates came from three countries: Mexico (26.3%), Argentina (22.6%) and Colombia (14.7%). The numbers of centers that participated in each country were as follows: Mexico, 16; Colombia, 14; Argentina, 12; Chile, 6; Venezuela, 6; Brazil, 4; Guatemala, 4; Honduras, 2; Panama, 2. Four countries submitted isolates in ≤2 of the 12 years of study (El Salvador 2009, 2010; Nicaragua 2006, 2007; Jamaica 2006; Puerto Rico 2006) and so are not included in the country by country analysis. They are included the analysis of data for Latin America as a whole. The number of isolates of each organism, by year, are shown in Additional file 1: Table S1, S2.

**Table 1:** Number of isolates collected by year from T.E.S.T. Latin America centers, 2004–2015

Country	Number of isolates^a^													
	2004	2005	2006	2007	2008	2009	2010	2011	2012	2013	2014	2015	2011–2015	2004–2015
Central America														
Guatemala	4	0	172	213	187	531	562	1	0	0	0	159	160	1829
Honduras	0	0	93	97	0	244	1	0	0	0	0	0	0	435
Panama	0	1	182	90	205	185	182	0	189	196	195	195	775	1620
Rest of Latin America														
Argentina	450	1064	612	1142	1402	1113	900	199	0	0	0	332	531	7214
Brazil	83	291	161	236	482	583	20	0	0	0	8	344	352	2208
Chile	5	228	318	624	446	359	0	0	61	197	217	330	805	2785
Colombia	0	76	461	122	1176	1072	719	341	166	189	196	182	1074	4700
Mexico	0	105	1111	1010	1921	1586	1242	94	182	456	193	513	1438	8413
Venezuela	1	0	181	358	240	574	200	137	0	0	0	318	455	2009
All Latin America^b^	543	1765	3661	3899	6059	6434	3982	772	598	1038	809	2373	5590	31,933

^a^Not all countries in Latin America participated in T.E.S.T. every year

^b^Includes all countries in Latin America that participated in T.E.S.T. Individual data for El Salvador, Nicaragua, Jamaica and Puerto Rico not present as contributed isolates in ≤2 years

### Gram-positive organisms

**Fig. 1:**
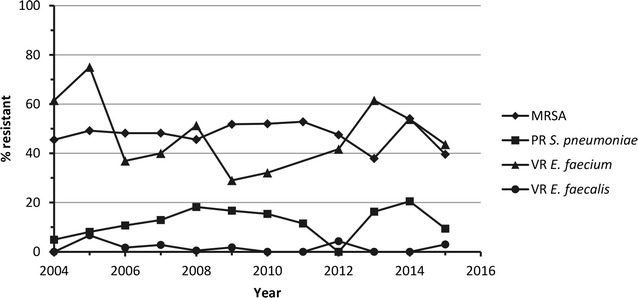
Rates of Gram-positive resistant phenotypes collected from Latin America by year, 2004–2015. MRSA N values: 2004, 30/66; 2005, 131/266; 2006, 247/512; 2007, 258/535; 2008, 374/821; 2009, 479/924; 2010, 325/625; 2011, 57/108; 2012, 47/99; 2013, 55/145; 2014, 60/111; 2015, 139/351. PR *S. pneumoniae* N values: 2004, 2/41; 2005, 10/123; 2006, 19/178; 2007, 30/232; 2008, 49/269; 2009, 43/258; 2010, 14/91; 2011, 6/52; 2012, 0/14; 2013, 8/49; 2014, 9/44; 2015, 8/85. VR *E. faecium* N values: 2004, 8/13; 2005, 9/12; 2006, 24/65; 2007, 22/55; 2008, 60/117; 2009, 40/138; 2010, 25/78; 2012, 5/12; 2013, 16/26; 2014, 7/13; 2015, 17/39. Data point for VR *E. faecium* for 2011 omitted as N < 10. VR *E. faecalis* N values: 2004, 0/25; 2005, 7/104; 2006, 4/231; 2007, 6/216; 2008, 2/404; 2009, 7/389; 2010, 0/258; 2011, 0/40; 2012, 2/46; 2013, 0/71; 2014, 0/56; 2015, 5/164.* MRSA* methicillin-resistant* S. aureus*,* PR* penicillin-resistant,* VR* vancomycin-resistant

Data on rates of Gram-positive resistant phenotypes of *S. aureus, S. pneumoniae, Enterococcus faecium* and *Enterococcus faecalis* are presented by country in Table 2 and by year in Fig. 1. Pooled (2004–2015) antimicrobial susceptibility data for these organisms, as well as *Streptococcus agalactiae*, are presented in Table 3, and year by year susceptibility data are presented in Additional file 1: Table S1.

**Table 2:** Rates of Gram-positive resistant phenotypes collected from Latin America by country, 2004–2015

Country	Methicillin-resistant *S. aureus*		Penicillin-resistant *S. pneumoniae*		Vancomycin-resistant *E. faecium*		Vancomycin-resistant *E. faecalis*	
	n/N	%	n/N	%	n/N	%	n/N	%
Central America								
Guatemala	226/336	67.3	0/15	0.0	6/29	20.7	0/116	0.0
Honduras	30/62	48.4	2/10	20.0	1/10	10.0	0/31	0.0
Panama	89/224	39.7	11/108	10.2	2/11	18.2	0/114	0.0
Rest of Latin America								
Argentina	454/947	47.9	35/412	8.5	60/115	52.2	2/408	0.5
Brazil	141/290	48.6	12/112	10.7	34/44	77.3	19/133	14.3
Chile	254/410	62.0	24/146	16.4	33/59	55.9	4/163	2.5
Colombia	261/653	40.0	25/187	13.4	33/86	38.4	5/314	1.6
Mexico	547/1243	44.0	73/350	20.9	55/175	31.4	2/565	0.4
Venezuela	136/265	51.3	14/81	17.3	9/38	23.7	1/121	0.8
All Latin America^a^	2202/4563	48.3	198/1436	13.8	235/576	40.8	33/2004	1.6

^a^Includes all countries in Latin America that participated in T.E.S.T. Individual data for El Salvador, Nicaragua, Jamaica and Puerto Rico not present as contributed isolates in ≤2 years

**Table 3:** Antimicrobial activity among Gram-positive organisms collected in Latin America, 2004–2015

Species (no. isolates) and antimicrobial agent	MIC (mg/L)			Susceptibility	
	MIC_50_	MIC_90_	MIC range	% S	% R
*Staphylococcus aureus* (4563)					
Amoxicillin–clavulanic acid	2	≥16	≤0.03 to ≥16	–	–
Ampicillin	16	≥32	≤0.06 to ≥32	–	–
Ceftriaxone	8	≥128	≤0.03 to ≥128	–	–
Levofloxacin	0.25	32	≤0.06 to ≥64	58.2	39.8
Linezolid	2	4	≤0.5 to 4	100	0.0
Meropenem (N = 3998)^a^	0.5	≥32	≤0.12 to ≥32	–	–
Minocycline	≤0.25	1	≤0.25 to ≥16	97.6	0.8
Penicillin	≥16	≥16	≤0.06 to ≥16	5.5	94.5
Piperacillin–tazobactam	2	≥32	≤0.25 to ≥32	–	–
Tigecycline	0.12	0.25	≤0.008 to 2	99.9	–
Vancomycin	1	1	≤0.12 to 2	100	0.0
*Staphylococcus aureus*, methicillin-resistant (2202/4563)					
Levofloxacin	8	32	≤0.06 to ≥64	20.3	77.4
Linezolid	2	2	≤0.5 to 4	100	0.0
Minocycline	≤0.25	1	≤0.25 to ≥16	96.2	1.3
Tigecycline	0.12	0.25	≤0.008 to 2	99.9	–
Vancomycin	1	1	≤0.12 to 2	100	0.0
*Streptococcus pneumoniae* (1436)					
Amoxicillin–clavulanic acid	≤0.03	2	≤0.03 to ≥16	94.6	2.0
Ampicillin (N = 1434)	≤0.06	2	≤0.06 to ≥32	–	–
Azithromycin (N = 1247)	0.12	64	≤0.03 to ≥128	72.6	26.9
Ceftriaxone	0.06	1	≤0.03 to ≥128	94.9	1.0
Clarithromycin (N = 1247)	0.03	64	≤0.015 to ≥128	72.7	26.8
Clindamycin (N = 1247)	0.06	64	≤0.015 to ≥128	88.0	11.9
Erythromycin (N = 1247)	0.06	64	≤0.015 to ≥128	71.7	27.3
Levofloxacin	1	1	≤0.06 to 32	98.9	0.3
Linezolid	1	1	≤0.5 to 2	100	–
Meropenem (N = 1256)^a^	≤0.12	0.5	≤0.12 to 16	79.6	7.7
Minocycline	1	8	≤0.25 to ≥16	59.8	28.8
Penicillin	≤0.06	2	≤0.06 to ≥16	54.7	13.8
Piperacillin–tazobactam	≤0.25	2	≤0.25 to ≥32	–	–
Tigecycline	0.015	0.06	≤0.008 to 0.5	95.5	–
Vancomycin	0.25	0.5	≤0.12 to 1	100	–
*Streptococcus pneumoniae*, penicillin-resistant (198/1436)					
Amoxicillin–clavulanic acid	2	8	≤0.03 to ≥16	63.6	13.1
Ampicillin (N = 197)	4	8	0.12 to ≥32	–	–
Azithromycin (N = 179)	16	≥128	≤0.03 to ≥128	41.3	58.1
Ceftriaxone	1	2	≤0.03 to ≥128	69.2	6.1
Clarithromycin (N = 179)	4	≥128	≤0.015 to ≥128	41.3	58.7
Clindamycin (N = 179)	0.12	≥128	≤0.015 to ≥128	62.0	38.0
Erythromycin (N = 179)	8	≥128	≤0.015 to ≥128	41.3	58.7
Levofloxacin	1	1	0.25 to 4	98.0	0.0
Linezolid	1	1	≤0.5 to 2	100	–
Meropenem (N = 184)^a^	0.5	1	≤0.12 to 16	7.6	43.5
Minocycline	4	≥16	≤0.25 to ≥16	32.8	55.6
Piperacillin–tazobactam	4	8	0.5 to ≥32	–	–
Tigecycline	0.015	0.03	≤0.008 to 0.5	98.0	–
Vancomycin	0.5	0.5	≤0.12 to 1	100	–
*Streptococcus agalactiae* (1339)					
Amoxicillin–clavulanic acid	0.06	0.12	≤0.03 to ≥16	–	–
Ampicillin	≤0.06	0.12	≤0.06 to 0.25	100	–
Ceftriaxone	0.06	0.12	≤0.03 to 2	99.8	–
Levofloxacin	0.5	1	≤0.06 to ≥64	97.7	1.7
Linezolid	1	1	≤0.5 to 2	100	–
Meropenem (N = 1198)^a^	≤0.12	≤0.12	≤0.12 to 0.5	100	–
Minocycline	8	≥16	≤0.25 to ≥16	27.6	61.8
Penicillin	≤0.06	0.12	≤0.06 to 0.12	100	–
Piperacillin–tazobactam	≤0.25	0.5	≤0.25 to ≥32	–	–
Tigecycline	0.03	0.06	≤0.008 to 0.5	99.9	–
Vancomycin	0.5	0.5	≤0.12 to 1	100	–
*Enterococcus faecium* (576)					
Amoxicillin–clavulanic acid	≥16	≥16	≤0.03 to ≥16	–	–
Ampicillin	≥32	≥32	≤0.06 to ≥32	26.0	74.0
Ceftriaxone	≥128	≥128	≤0.03 to ≥128	–	–
Levofloxacin	≥64	≥64	≤0.06 to ≥64	21.7	71.0
Linezolid	2	2	≤0.5 to 4	99.8	0.0
Meropenem (N = 524)^a^	≥32	≥32	≤0.12 to ≥32	–	–
Minocycline	2	≥16	≤0.25 to ≥16	62.0	19.8
Penicillin	≥16	≥16	≤0.06 to ≥16	22.6	77.4
Piperacillin–tazobactam	≥32	≥32	≤0.25 to ≥32	–	–
Tigecycline	0.06	0.25	≤0.008 to 1	99.5	–
Vancomycin	2	≥64	≤0.12 to ≥64	56.8	40.8
*Enterococcus faecium*, vancomycin-resistant (235/576)					
Amoxicillin–clavulanic acid	≥16	≥16	1 to ≥16	–	–
Ampicillin	≥32	≥32	2 to ≥32	0.9	99.1
Ceftriaxone	≥128	≥128	4 to ≥128	–	–
Levofloxacin	≥64	≥64	2 to ≥64	0.9	97.0
Linezolid	2	2	≤0.5 to 2	100	0.0
Meropenem (N = 213)^a^	≥32	≥32	≤0.12 to ≥32	–	–
Minocycline	≤0.25	≥16	≤0.25 to ≥16	71.5	17.0
Penicillin	≥16	≥16	4 to ≥16	1.3	98.7
Piperacillin–tazobactam	≥32	≥32	2 to ≥32	–	–
Tigecycline	0.06	0.25	≤0.008 to 1	98.7	–
*Enterococcus faecalis* (2004)					
Amoxicillin–clavulanic acid	0.5	1	≤0.03 to ≥16	–	–
Ampicillin	1	2	≤0.06 to ≥32	99.0	1.0
Ceftriaxone	≥128	≥128	≤0.03 to ≥128	–	–
Levofloxacin	1	≥64	≤0.06 to ≥64	69.4	29.1
Linezolid	2	2	≤0.5 to 4	99.8	0.0
Meropenem (N = 1771)^a^	4	8	≤0.12 to ≥32	–	–
Minocycline	8	≥16	≤0.25 to ≥16	34.9	30.4
Penicillin	2	4	≤0.06 to ≥16	98.4	1.6
Piperacillin–tazobactam	4	8	≤0.25 to ≥32	–	–
Tigecycline	0.12	0.25	≤0.008 to 1	99.7	–
Vancomycin	1	2	≤0.12 to ≥64	98.1	1.6
*Enterococcus faecalis*, vancomycin-resistant (33/2004)					
Amoxicillin–clavulanic acid	1	≥16	0.25 to ≥16	–	–
Ampicillin	2	≥32	0.5 to ≥32	78.8	21.2
Ceftriaxone	≥128	≥128	128 to ≥128	–	–
Levofloxacin	32	≥64	1 to ≥64	6.1	93.9
Linezolid	1	2	1 to 2	100	0.0
Meropenem (N = 27)^a^	16	≥32	2 to ≥32	–	–
Minocycline	4	≥16	≤0.25 to ≥16	51.5	18.2
Penicillin	8	≥16	2 to ≥16	75.8	24.2
Piperacillin–tazobactam	8	≥32	2 to ≥32	–	–
Tigecycline	0.12	0.25	0.015 to 0.25	100	–

–, no CLSI breakpoints available

*MIC* minimum inhibitory concentration, *MIC*_*50*_ MIC required to inhibit growth of 50% of isolates, *MIC*_*90*_ MIC required to inhibit growth of 90% of isolates, *S* susceptible, *R* resistant

^a^Susceptibility data for imipenem were collected from 2004 to 2006, after which time imipenem was replaced by meropenem

A total of 4563 isolates of *S. aureus* were collected in Latin America between 2004 and 2015, and almost half (48.3%) were MRSA (Table 2). Rates of MRSA were highest in Guatemala and Chile (67.3 and 62.0%, respectively) and lowest in Panama and Colombia (39.7 and 40.0%, respectively). MRSA rates appeared stable, although some variability occurred in the more recent years [between 2004 and 2015 rates were lowest in 2013 (37.9%; 55/145) and highest in 2014 (54.1%; 60/111)] (Fig. 1). All *S. aureus* isolates, including MRSA isolates, were susceptible to linezolid and vancomycin (Table 3). Susceptibility rates among all *S. aureus* to tigecycline and minocycline were 99.9 and 97.6%, respectively (Table 3). Among MRSA isolates the rates of susceptibility to tigecycline and minocycline were 99.9 and 96.2%, respectively. Rates of susceptibility were stable over time against both *S. aureus* and MRSA (Additional file 1: Table S1). One exception was levofloxacin, susceptibility to which increased over the course of the study for all *S. aureus* isolates [56.1% (37/66) in 2004 and 74.9% (263/351) in 2015] and for MRSA isolates [3.3% (1/30) in 2004 and 46.8% (65/139) in 2015] (Additional file 1: Table S1).

Over the 2004–2015 time period, 1436 isolates of *S. pneumoniae* were submitted, of which 13.8% were penicillin-resistant (Table 2). Resistance to penicillin ranged from 0% (0/15) in Guatemala to 20.9% (73/350) in Mexico. Rates of penicillin resistance were ≤21.0% between 2004 and 2015, and ranged from 0% (0/14) in 2012 to 20.5% (9/44) in 2014 (Fig. 1). The number of penicillin-resistant isolates was ≤10 for seven years of the study (2004, 2005 and 2011–2015). All *S. pneumoniae* isolates were susceptible to linezolid and vancomycin (Table 3). Susceptibility rates for all *S. pneumoniae* isolates were ≥94.0% for levofloxacin, tigecycline, ceftriaxone and amoxicillin–clavulanic acid. Year by year data shows susceptibility rates were stable for levofloxacin (≥97.0% in all years) and ceftriaxone (≥89.0% in all years), however susceptibility rates were more variable for the other agents on the panel (Additional file 1: Table S1). All *S. pneumoniae* isolates were susceptible to tigecycline between 2010 and 2015, prior to that susceptibility increased from 78.0% (32/41) in 2004 to 99.2% (256/258) in 2009. Conversely, susceptibility to minocycline decreased between 2004 and 2009 [from 92.7% (38/41) to 32.2% (83/258)], and higher rates of susceptibility were reported in all subsequent years, with a rate in 2015 of 71.8% (61/85) (Additional file 1: Table S1). Susceptibility to levofloxacin and tigecycline among penicillin-resistant isolates was also high (98.0% for each for the 2004–2015 pooled time period); however, susceptibility to ceftriaxone and amoxicillin–clavulanic acid among penicillin-resistant isolates was reduced (69.2 and 63.6%, respectively) (Table 3).

A total of 1339 isolates of *S. agalactiae* were submitted to T.E.S.T. between 2004 and 2015 in Latin America (Table 3). Susceptibility to the majority of agents was unchanged over the course of the study (Additional file 1: Table S1) and all isolates were susceptible to ampicillin, linezolid, meropenem, penicillin and vancomycin (Table 3). More than 97.0% were susceptible to tigecycline, ceftriaxone and levofloxacin; however, susceptibility to minocycline was lower (27.6%) and variable over the course of the study (Table 3; Additional file 1: Table S1).

A total of 576 isolates of *E. faecium* were collected in Latin America between 2004 and 2015, and vancomycin resistance was seen in 40.8% (Table 2). Rates of vancomycin resistance among *E. faecium* isolates were highest in Brazil (77.3%), Chile (55.9%) and Argentina (52.2%). Rates of vancomycin resistance among *E. faecium* isolates were lower in countries in Central America (Guatemala, Honduras and Panama) than in the rest of the Latin America. Vancomycin resistance rates were variable over the course of the study (Fig. 1). High percentages (>99.0%) of *E. faecium* isolates were susceptible to linezolid and tigecycline (Table 3) and rates were unchanged over the course of the study (Additional file 1: Table S1). A single *E. faecium* isolate was non-susceptible to linezolid. Among the vancomycin-resistant isolates, all were susceptible to linezolid and 98.7% were susceptible to tigecycline (Table 3).

Of the 2004 *E. faecalis* isolates submitted between 2004 and 2015, 1.6% were vancomycin-resistant (Table 2). Rates of vancomycin resistance were ≤2.5% in all countries except Brazil, which had a resistance rate of 14.3%. None of the *E. faecalis* isolates submitted by Central American countries were resistant to vancomycin. No vancomycin-resistant *E. faecalis* isolates were collected in 2004, 2010, 2011, 2013 or 2014, and less than 10 isolates were collected for any other year (Additional file 1: Table S1). Susceptibility rates for all *E. faecalis* isolates were >98.0% for linezolid, tigecycline, ampicillin, penicillin and vancomycin (Table 3) and were unchanged over time (Additional file 1: Table S1). This high level of susceptibility to linezolid and tigecycline was maintained among vancomycin-resistant isolates, whereas susceptibility to ampicillin and penicillin decreased to 78.8 and 75.8%, respectively (Table 3). Susceptibility among all *E. faecalis* isolates to minocycline decreased from 56.0% (14/25) in 2004 to 20.0% (8/40) in 2011; rates after 2011 were variable but did show a trend towards increasing susceptibility (Additional file 1: Table S1).

### Gram-negative organisms

**Fig. 2:**
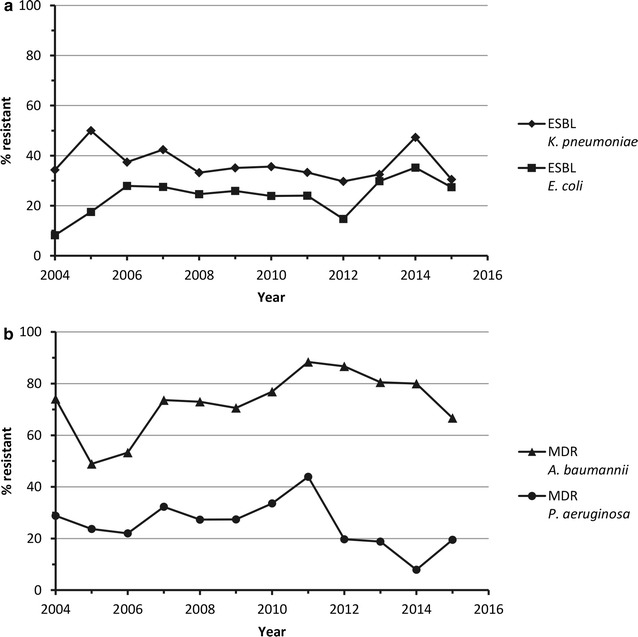
Rates of Gram-negative resistant phenotypes collected from Latin America by year, 2004–2015. **a** ESBL *K. pneumoniae* N values: 2004, 23/67; 2005, 101/202; 2006, 166/444; 2007, 196/462; 2008, 263/791; 2009, 299/851; 2010, 187/525; 2011, 29/87; 2012, 22/74; 2013, 38/117; 2014, 43/91; 2015, 98/321. ESBL *E. coli* N values: 2004, 6/73; 2005, 37/211; 2006, 164/588; 2007, 147/534; 2008, 220/893; 2009, 272/1050; 2010, 158/660; 2011, 30/125; 2012, 15/102; 2013, 45/151; 2014, 37/105; 2015, 115/420. **b** MDR *A. baumannii* N values: 2004, 40/54; 2005, 66/135; 2006, 131/246; 2007, 209/284; 2008, 343/470; 2009, 344/487; 2010, 240/312; 2011, 38/43; 2012, 39/45; 2013, 62/77; 2014, 48/60; 2015, 94/141. MDR *P. aeruginosa* N values: 2004, 17/59; 2005, 40/169; 2006, 94/427; 2007, 124/384; 2008, 200/732; 2009, 206/753; 2010, 161/479; 2011, 29/66; 2012, 13/66; 2013, 22/117; 2014, 7/89; 2015, 53/272. ESBL-producing *K. oxytoca*_-_ and β-lactamase positive *H. influenzae* are not shown due to low number of isolates year on year. *ESBL* extended-spectrum β-lactamase, *MDR* multidrug-resistant

Data on rates of Gram-negative resistant phenotypes of *K. pneumoniae*, *Klebsiella oxytoca*, *E. coli*, *P. aeruginosa*, *A. baumannii* and *H. influenzae* are presented by country in Table 4, and by year in Fig. 2 (with the exception of *K. oxytoca* and *H. influenzae).* Antimicrobial susceptibility data for these organisms, as well as *Enterobacter* spp. and *Serratia marcescens*, are presented in Table 5, and year by year susceptibility data are presented in Additional file 1: Table S2.

**Table 4:** Rates of Gram-negative resistant phenotypes collected from Latin America by country, 2004–2015

Country	ESBL-producing *K. pneumoniae*		ESBL-producing *K. oxytoca*		ESBL-producing *E. coli*		βLPos *H. influenzae*		MDR *A. baumannii*		MDR *P. aeruginosa*	
	n/N	%	n/N	%	n/N	%	n/N	%	n/N	%	n/N	%
Central America												
Guatemala	153/253	60.5	3/8	37.5	163/414	39.4	2/16	12.5	150/189	79.4	103/235	43.8
Honduras	56/76	73.7	1/1	100	31/78	39.7	2/8	25.0	39/51	76.5	15/47	31.9
Panama	79/210	37.6	2/4	50.0	49/225	21.8	12/78	15.4	95/122	77.9	21/176	11.9
Rest of Latin America												
Argentina	367/869	42.2	14/83	16.9	133/949	14.0	109/468	23.3	465/573	81.2	192/749	25.6
Brazil	122/270	45.2	3/26	11.5	51/300	17.0	19/96	19.8	148/174	85.1	73/232	31.5
Chile	216/341	63.3	6/34	17.6	130/386	33.7	31/130	23.8	145/217	66.8	81/286	28.3
Colombia	138/593	23.3	9/80	11.3	92/708	13.0	9/168	5.4	180/319	56.4	82/535	15.3
Mexico	241/1025	23.5	23/152	15.1	510/1405	36.3	65/232	28.0	297/518	57.3	283/1035	27.3
Venezuela	50/273	18.3	4/17	23.5	54/296	18.2	18/89	20.2	101/137	73.7	77/236	32.6
All Latin America^a^	1465/4032	36.3	67/409	16.4	1246/4912	25.4	270/1300	20.8	1654/2354	70.3	966/3613	26.7

*ESBL* extended-spectrum β-lactamase, *βLPos* β-lactamase positive, *MDR* multidrug-resistant

^a^Includes all countries in Latin America that participated in T.E.S.T. Individual data for El Salvador, Nicaragua, Jamaica and Puerto Rico not present as contributed isolates in ≤2 years

**Table 5:** Antimicrobial activity among Gram-negative organisms collected in Latin America, 2004–2015

Species (no. isolates) and antimicrobial agent	MIC (mg/L)			Susceptibility	
	MIC_50_	MIC_90_	MIC range	% S	% R
*Klebsiella pneumoniae* (4032)					
Amikacin	2	32	≤0.5 to ≥128	86.9	8.9
Amoxicillin–clavulanic acid	16	≥64	≤0.12 to ≥64	47.3	35.3
Ampicillin (N = 4024)	≥64	≥64	1 to ≥64	1.3	92.8
Cefepime	1	≥64	≤0.5 to ≥64	55.8	33.9
Ceftriaxone	2	≥128	≤0.06 to ≥128	49.1	49.7
Levofloxacin	0.25	≥16	≤0.008 to ≥16	65.3	31.6
Meropenem (N = 3555)^a^	≤0.06	1	≤0.06 to ≥32	90.9	7.3
Minocycline	4	≥32	≤0.5 to ≥32	61.4	25.2
Piperacillin–tazobactam	4	≥256	≤0.06 to ≥256	64.0	25.4
Tigecycline	0.5	2	≤0.008 to ≥32	95.7	0.9
*Klebsiella pneumoniae*, ESBL (1465/4032)					
Amikacin	8	≥128	≤0.5 to ≥128	75.3	16.4
Amoxicillin–clavulanic acid	32	≥64	≤0.12 to ≥64	12.8	57.7
Ampicillin	≥64	≥64	4 to ≥64	0.1	99.6
Cefepime	32	≥64	≤0.5 to ≥64	11.2	71.3
Ceftriaxone	≥128	≥128	≤0.06 to ≥128	1.0	97.8
Levofloxacin	8	≥16	≤0.008 to ≥16	39.4	55.6
Meropenem (N = 1270)^a^	≤0.06	4	≤0.06 to ≥32	86.9	10.2
Minocycline	4	≥32	≤0.5 to ≥32	51.6	31.5
Piperacillin–tazobactam	64	≥256	0.12 to ≥256	35.5	45.1
Tigecycline	0.5	2	0.03 to 16	93.4	1.4
*Klebsiella oxytoca* (409)					
Amikacin	2	8	≤0.5 to ≥128	95.6	2.9
Amoxicillin–clavulanic acid	4	32	0.25 to ≥64	70.4	17.6
Ampicillin	≥64	≥64	≤0.5 to ≥64	1.7	90.6
Cefepime	≤0.5	16	≤0.5 to ≥64	79.5	13.0
Ceftriaxone	0.12	≥128	≤0.06 to ≥128	69.7	27.6
Levofloxacin	0.06	≥16	≤0.008 to ≥16	82.9	15.6
Meropenem (N = 333)^a^	≤0.06	0.12	≤0.06 to 16	97.6	1.5
Minocycline	2	16	≤0.5 to ≥32	79.0	11.2
Piperacillin–tazobactam	2	64	≤0.06 to ≥256	84.6	9.8
Tigecycline	0.25	1	0.06 to 4	98.0	0.0
*Klebsiella oxytoca*, ESBL (67/409)					
Amikacin	4	32	≤0.5 to ≥128	89.6	6.0
Amoxicillin–clavulanic acid	16	≥64	0.25 to ≥64	26.9	35.8
Ampicillin	≥64	≥64	32 to ≥64	0.0	100
Cefepime	8	≥64	≤0.5 to ≥64	26.9	49.3
Ceftriaxone	64	≥128	≤0.06 to ≥128	3.0	91.0
Levofloxacin	2	≥16	0.03 to ≥16	55.2	41.8
Meropenem (N = 48)^a^	≤0.06	0.25	≤0.06 to 16	95.8	4.2
Minocycline	8	≥32	≤0.5 to ≥32	49.3	22.4
Piperacillin–tazobactam	8	≥256	≤0.06 to ≥256	61.2	22.4
Tigecycline	0.5	2	0.06 to 4	94.0	0.0
*Escherichia coli* (4912)					
Amikacin	2	8	≤0.5 to ≥128	95.8	2.2
Amoxicillin–clavulanic acid	8	32	≤0.12 to ≥64	51.7	22.0
Ampicillin	≥64	≥64	≤0.5 to ≥64	21.2	77.7
Cefepime	≤0.5	≥64	≤0.5 to ≥64	67.6	23.2
Ceftriaxone	0.12	≥128	≤0.06 to ≥128	60.2	37.9
Levofloxacin	4	≥16	≤0.008 to ≥16	47.8	48.8
Meropenem (N = 4284)^a^	≤0.06	0.12	≤0.06 to ≥32	98.1	1.3
Minocycline	4	≥32	≤0.5 to ≥32	61.9	24.0
Piperacillin–tazobactam	2	32	≤0.06 to ≥256	85.7	6.9
Tigecycline	0.25	0.5	≤0.008 to ≥32	99.7	<0.1
*Escherichia coli*, ESBL (1246/4912)					
Amikacin	4	16	≤0.5 to ≥128	90.6	4.5
Amoxicillin–clavulanic acid	16	32	0.25 to ≥64	23.4	34.1
Ampicillin	≥64	≥64	1 to ≥64	0.7	99.0
Cefepime	32	≥64	≤0.5 to ≥64	11.0	71.6
Ceftriaxone	≥128	≥128	≤0.06 to ≥128	1.3	97.0
Levofloxacin	≥16	≥16	0.015 to ≥16	11.8	84.4
Meropenem (N = 1103)^a^	≤0.06	0.12	≤0.06 to ≥32	97.0	2.2
Minocycline	4	≥32	≤0.5 to ≥32	55.4	30.2
Piperacillin–tazobactam	8	128	≤0.06 to ≥256	74.4	10.6
Tigecycline	0.25	0.5	≤0.008 to 4	99.8	0.0
*Enterobacter* spp. (3818)					
Amikacin	2	16	≤0.5 to ≥128	90.2	5.9
Amoxicillin–clavulanic acid	≥64	≥64	≤0.12 to ≥64	5.1	91.8
Ampicillin (N = 3810)	≥64	≥64	≤0.5 to ≥64	3.7	90.0
Cefepime	≤0.5	≥64	≤0.5 to ≥64	70.5	17.6
Ceftriaxone	0.5	≥128	≤0.06 to ≥128	53.5	43.8
Levofloxacin	0.12	≥16	≤0.008 to ≥16	78.4	18.6
Meropenem (N = 3320)^a^	≤0.06	0.5	≤0.06 to ≥32	95.1	3.3
Minocycline	4	≥32	≤0.5 to ≥32	63.4	19.6
Piperacillin–tazobactam	4	≥256	≤0.06 to ≥256	71.9	16.8
Tigecycline	0.5	2	≤0.008 to ≥32	95.7	0.6
*Serratia marcescens* (1577)					
Amikacin	2	32	≤0.5 to ≥128	83.4	9.1
Amoxicillin–clavulanic acid	≥64	≥64	≤0.12 to ≥64	4.9	91.8
Ampicillin (N = 1575)	≥64	≥64	≤0.5 to ≥64	2.8	90.2
Cefepime	≤0.5	32	≤0.5 to ≥64	76.0	16.5
Ceftriaxone	0.5	≥128	≤0.06 to ≥128	67.1	29.7
Levofloxacin	0.25	8	≤0.008 to ≥16	84.9	10.6
Meropenem (N = 1347)^a^	≤0.06	0.5	≤0.06 to ≥32	95.0	3.7
Minocycline	4	16	≤0.5 to ≥32	64.1	14.8
Piperacillin–tazobactam	2	128	≤0.06 to ≥256	83.4	10.1
Tigecycline	1	2	≤0.008 to 16	94.8	0.8
*Pseudomonas aeruginosa* (3613)					
Amikacin	4	≥128	≤0.5 to ≥128	72.8	20.1
Amoxicillin–clavulanic acid	≥64	≥64	0.5 to ≥64	–	–
Ampicillin	≥64	≥64	1 to ≥64	–	–
Cefepime	8	≥64	≤0.5 to ≥64	60.4	25.4
Ceftazidime	8	≥64	≤1 to ≥64	56.8	33.4
Ceftriaxone	64	≥128	≤0.06 to ≥128	–	–
Levofloxacin	2	≥16	0.015 to ≥16	53.5	40.0
Meropenem (N = 3151)^a^	2	≥32	≤0.06 to ≥32	53.8	36.9
Minocycline	≥32	≥32	≤0.5 to ≥32	–	–
Piperacillin–tazobactam	16	≥256	≤0.06 to ≥256	58.5	24.4
Tigecycline	8	16	≤0.008 to ≥32	–	–
*Pseudomonas aeruginosa,* MDR (966/3613)					
Amikacin	64	≥128	≤0.5 to ≥128	24.5	66.7
Amoxicillin–clavulanic acid	≥64	≥64	4 to ≥64	–	–
Ampicillin	≥64	≥64	1 to ≥64	–	–
Cefepime	≥64	≥64	≤0.5 to ≥64	8.8	72.6
Ceftazidime	32	≥64	2 to ≥64	8.7	81.5
Ceftriaxone	≥128	≥128	4 to ≥128	–	–
Levofloxacin	≥16	≥16	0.25 to ≥16	2.2	95.4
Meropenem (N = 861)^a^	≥32	≥32	≤0.06 to ≥32	4.6	90.0
Minocycline	≥32	≥32	≤0.5 to ≥32	–	–
Piperacillin–tazobactam	128	≥256	0.5 to ≥256	11.2	62.7
Tigecycline	16	≥32	0.25 to ≥32	–	–
*Acinetobacter baumannii* (2354)					
Amikacin	64	≥128	≤0.5 to ≥128	30.8	55.9
Amoxicillin–clavulanic acid	≥64	≥64	0.25 to ≥64	–	–
Ampicillin	≥64	≥64	≤0.5 to ≥64	–	–
Cefepime	32	≥64	≤0.5 to ≥64	22.0	65.2
Ceftazidime	32	≥64	≤1 to ≥64	17.2	74.9
Ceftriaxone	≥128	≥128	≤0.06 to ≥128	9.1	79.4
Levofloxacin	8	≥16	≤0.008 to ≥16	19.3	69.4
Meropenem (N = 2046)^a^	≥32	≥32	≤0.06 to ≥32	25.7	69.9
Minocycline	≤0.5	8	≤0.5 to ≥32	88.3	6.7
Piperacillin–tazobactam	≥256	≥256	≤0.06 to ≥256	17.2	74.8
Tigecycline	0.5	2	≤0.008 to ≥32	–	–
*Acinetobacter baumannii,* MDR (1654/2354)					
Amikacin	≥128	≥128	≤0.5 to ≥128	11.5	77.1
Amoxicillin–clavulanic acid	≥64	≥64	8 to ≥64	–	–
Ampicillin	≥64	≥64	≤0.5 to ≥64	–	–
Cefepime	≥64	≥64	≤0.5 to ≥64	5.3	82.3
Ceftazidime	≥64	≥64	≤1 to ≥64	4.3	89.8
Ceftriaxone	≥128	≥128	0.25 to ≥128	0.4	95.1
Levofloxacin	≥16	≥16	0.03 to ≥16	1.6	90.7
Meropenem (N = 1493)^a^	≥32	≥32	≤0.06 to ≥32	6.8	89.8
Minocycline	1	8	≤0.5 to ≥32	86.2	8.4
Piperacillin–tazobactam	≥256	≥256	≤0.06 to ≥256	1.7	93.3
Tigecycline	0.5	2	0.03 to ≥32	–	–
*Haemophilus influenzae* (1300)					
Amikacin (N = 1299)	4	8	≤0.5 to ≥128	–	–
Amoxicillin–clavulanic acid	0.5	2	≤0.12 to ≥64	99.4	0.6
Ampicillin	≤0.5	32	≤0.5 to ≥64	77.8	19.5
Cefepime	≤0.5	≤0.5	≤0.5 to 16	99.3	–
Ceftriaxone	≤0.06	≤0.06	≤0.06 to 32	99.7	–
Levofloxacin	0.015	0.03	≤0.008 to 2	100	–
Meropenem (N = 1075)^a^	≤0.06	0.12	≤0.06 to 0.5	100	–
Minocycline (N = 1299)	≤0.5	1	≤0.5 to 16	98.8	0.5
Piperacillin–tazobactam	≤0.06	≤0.06	≤0.06 to 16	99.3	0.7
Tigecycline	0.12	0.25	≤0.008 to 2	97.8	–
*Haemophilus influenzae*, βLPos (270/1300)					
Amikacin	4	8	≤0.5 to 16	–	–
Amoxicillin–clavulanic acid	1	2	≤0.12 to ≥64	98.5	1.5
Ampicillin	16	≥64	≤0.5 to ≥64	0.7	92.6
Cefepime	≤0.5	≤0.5	≤0.5 to 16	98.9	–
Ceftriaxone	≤0.06	≤0.06	≤0.06 to 16	99.6	–
Levofloxacin	0.015	0.03	≤0.008 to 0.5	100	–
Meropenem (N = 236)^a^	≤0.06	0.12	≤0.06 to 0.5	100	–
Minocycline	≤0.5	1	≤0.5 to 16	98.1	0.7
Piperacillin–tazobactam	≤0.06	≤0.06	≤0.06 to 16	99.6	0.4
Tigecycline	0.12	0.25	≤0.008 to 0.5	98.5	–

–, no CLSI breakpoints available

*MIC* minimum inhibitory concentration, *MIC*_*50*_ MIC required to inhibit growth of 50% of isolates, *MIC*_*90*_ MIC required to inhibit growth of 90% of isolates, *S* susceptible, *R* resistant, *ESBL* extended-spectrum β-lactamase, *βLPos* β-lactamase positive, *MDR* multidrug-resistant

^a^Susceptibility data for imipenem were collected from 2004 to 2006, after which time imipenem was replaced by meropenem

Among the 4032 *K. pneumoniae* isolates submitted between 2004 and 2015, 36.3% were ESBL-producers (Table 4) and rates of ESBL production ranged from 18.3% in Venezuela to 73.7% in Honduras. Figure 2a shows *K. pneumoniae* ESBL production rate was relatively stable for the 2004–2015 time period. Among *K. pneumoniae* isolates, susceptibility was highest to tigecycline, meropenem and amikacin (95.7, 90.9 and 86.9%, respectively); susceptibility among ESBL-producers was also highest to these agents (93.4, 86.9 and 75.3%, respectively) (Table 5). Susceptibility rates to tigecycline and meropenem were stable across the years of the study, whereas rates to amikacin were more variable (Additional file 1: Table S2). Among both all *K. pneumoniae* and ESBL-producers susceptibility to minocycline decreased from 2004 [80.6% (54/67) and 69.6% (16/23), respectively] until 2010 [44.4% (233/525) and 25.7% (48/187), respectively] and then increased, resulting in higher rates of susceptibility to minocycline in 2015 [81.9% (263/321) and 84.7% (83/98), respectively) (Additional file 1: Table S2). The susceptibility rate to levofloxacin among all *K. pneumoniae* isolates was 65.3%, and among ESBL-producing isolates was 39.4% (resistance rates 31.6 and 55.6%, respectively) (Table 5) and although there was some variability no trend was seen over time (Additional file 1: Table S2).

A total of 409 *K. oxytoca* isolates were collected, of which 16.4% were ESBL-producers (Table 4). Among all *K. oxytoca* isolates, susceptibility rates were highest to tigecycline, meropenem and amikacin (98.0, 97.6 and 95.6%, respectively) (Table 5) and little variability was seen over time (Additional file 1: Table S2). Numbers of ESBL-producing *K. oxytoca* were low in each year (≤14 isolates); in years with ≥10 isolates rates of susceptibility were highest to tigecycline, meropenem and amikacin (Additional file 1: Table S2).

Of the *E. coli* isolates collected, 25.4% were ESBL-producers and the percentage of isolates that produced ESBLs was highest in Honduras, Guatemala and Mexico (Table 4). Among all *E. coli* isolates, susceptibility was highest to tigecycline and meropenem (99.7 and 98.1%, respectively), and these rates were similar among the ESBL-producers (99.8 and 97.0%, respectively) (Table 5). Rates of susceptibility to tigecycline and meropenem were stable across the 2004–2015 time period (Additional file 1: Table S2). Susceptibility to minocycline decreased between 2004 and 2010/2011 and then increased, resulting in a similar rate of susceptibility in 2004 and 2015 [76.7% (56/73) and 81.7% (343/420), respectively] (Additional file 1: Table S2). For the other agents on the panel, susceptibility rates were lower among ESBL-producing *E. coli* compared with *E. coli* isolates overall. The rate of levofloxacin susceptibility among all *E. coli* isolates was 47.8%, and among ESBL-producing *E. coli* isolates was 11.8% (resistance rates were 48.8 and 84.4%, respectively). Susceptibility to meropenem was lower among *K. pneumoniae* isolates (90.9%) than *E. coli* isolates (98.1%).

*Enterobacte*r spp. and *S. marcescens* were highly susceptible to tigecycline (95.7 and 94.8%, respectively), meropenem (95.1 and 95.0%, respectively), and amikacin (90.2 and 83.4%, respectively) (Table 5), and rates were stable across the 2004–2015 time period (Additional file 1: Table S2).

Of the 3613 *P. aeruginosa* isolates submitted by Latin American centers between 2004 and 2015, 26.7% were MDR (Table 4). The countries that submitted the highest percentages of MDR *P. aeruginosa* isolates were Guatemala, Venezuela, Honduras and Brazil (43.8, 32.6, 31.9 and 31.5%, respectively). The year on year rates of MDR were variable across the 2004–2015 time period, however <20% of *P aeruginosa* were MDR between 2012 and 2015 (Fig. 2b). Breakpoints were available for six of the agents on the panel. Of these, the agents with the highest rate of susceptibility against *P. aeruginosa* was amikacin (72.8%) (Table 5). Susceptibility to amikacin increased between 2011 [59.1% (39/66)] and 2014 [92.1% (82/89)] although there was a small decrease in 2015 [83.8% (228/272)] (Additional file 1: Table S2). Among all *P. aeruginosa* isolates, 56.8% were susceptible to ceftazidime. Among isolates that were MDR, susceptibility for all agents was <25.0%. The meropenem susceptibility rate among all *P. aeruginosa* isolates was 53.8% (resistance rate 36.9%).

Over the 2004–2015 time period, 2354 *A. baumannii* isolates were submitted, and 70.3% were MDR (Table 4). By country Brazil and Argentina had the highest levels of MDR (85.1 and 81.2%, respectively). Figure 2b shows variability in rates of MDR among *A. baumannii* across the 2004–2015 time period; however, between 2011 and 2015 MDR rates decreased each year from 88.4% (38/43) in 2011 to 66.7% (94/141) in 2015. The agents with the lowest MIC_90_ values among all *A. baumannii* isolates were tigecycline and minocycline (2 and 8 mg/L, respectively); these values were the same among MDR *A. baumannii* isolates (Table 5). Among all *A. baumannii* isolates, 30.8% were sensitive to amikacin. Year on year data from 2006 onwards shows a trend towards decreasing susceptibility of *A. baumannii* to meropenem [from 34.7% (43/124) in 2006 to 20.6% (29/141) in 2015] (Additional file 1: Table S2). Over the course of the study rates of susceptibility to minocycline decreased from 98.1% (53/54) in 2004 to 83.0% (117/141) in 2015; however, susceptibility to amikacin increased reaching 50.4% (71/141) in 2015 (Additional file 1: Table S2). A similar pattern was seen among MDR *A. baumannii* (Additional file 1: Table S2).

Of the 1300 *H. influenzae* isolates submitted between 2004 and 2015, 20.8% were β-lactamase positive (Table 4). The country with the highest rate of β-lactamase positive isolates was Mexico (28.0%), whilst the lowest rate was in Colombia (5.4%). All *H. influenzae* isolates were susceptible to levofloxacin and meropenem (Table 5) and rates of susceptibility were consistent across the years of the study (Additional file 1: Table S2). Among all *H. influenzae* isolates and among β-lactamase positive isolates, susceptibility was ≥97.0% to ceftriaxone, amoxicillin–clavulanic acid, cefepime, piperacillin–tazobactam, minocycline and tigecycline.

## Discussion

This study reports on the rates of resistant phenotypes and in vitro antimicrobial susceptibility among important Gram-positive and Gram-negative isolates collected in Latin America between 2004 and 2015. It provides an update to previous publications which reported T.E.S.T. data from Latin America [9–11]. Tigecycline maintained its in vitro activity against the isolates collected in this study (susceptibility >93.0%, MIC_90_ 2 mg/L for *A. baumannii*). As previously reported, tigecycline was not active against *P. aeruginosa* [16].

Historically, the prevalence of MRSA has been reported to be increasing in the Latin American region. For example, the SENTRY study reported a significant increase in MRSA rates in Latin America between 1997 and 2006 (from 33.8 to 40.2%; p = 0.007) [17]. Previous T.E.S.T. reports have suggested a stabilization of rates [11] and this T.E.S.T. study of data for isolates collected between 2004 and 2015 continues to suggest that rates are stable in the region, although with country variations. The overall rate of MRSA in this study was 48.3%, which is similar to a SENTRY report from Latin America for the 2011–2014 time period (44.7%) [18]. Recent studies from Europe (between 2012 and 2015) and the USA (between 2005 and 2011) have reported decreasing rates of MRSA [19, 20]. Such reports suggest that global efforts regarding infection control and antimicrobial stewardships are having an impact.

Linezolid and vancomycin are key tools in the treatment of MRSA as infections are often caused by organisms resistant to other antimicrobials. As reported by other studies in Latin America [1, 3, 18, 21], all *S. aureus* isolates (including MRSA) collected as part of T.E.S.T between 2004 and 2015 were susceptible to linezolid and vancomycin. Small numbers of tigecycline non-susceptible isolates were collected in the early years of the T.E.S.T. program, as previously reported by Garza-González et al. [11]. However, from 2010 onwards all *S. aureus* isolates (including MRSA) were susceptible to tigecycline. This was also the case in the Latin American SENTRY study in which all *S. aureus* isolates (including MRSA) collected over a similar time (2011–2014) were susceptible to tigecycline [18].

Linezolid-resistant *Enterococcus* spp. have previously been reported in Latin America [18]. However, none of the *Enterococcus* spp. isolates submitted to T.E.S.T. between 2004 and 2015 were linezolid-resistant. There were five intermediate (MIC 4 mg/L) isolates: 1 *E. faecium* collected in Argentina in 2009 and 4 *E. faecalis*, 3 collected in Mexico in 2009 and 1 in El Salvador in 2010. The rate of vancomycin-resistant *E. faecium* was 40.8%, which was lower than reported by Sader et al. [18] for the 2011–2014 time period (50.3%). Year on year rates of vancomycin resistance in this study were variable, although this is likely to be in part due to the low number of isolates collected in some years. Interestingly, the rates of vancomycin-resistant *E. faecium* were lower in the Central American countries included in this study (Guatemala, Honduras and Panama) compared with the rest of Latin America, although it should be noted that a relatively low number of *E. faecium* isolates were collected in Central America. Sader et al. [18] also reported variable *E. faecium* vancomycin resistance rates (26.3% in Argentina to 71.7% in Brazil between 2011 and 2014), although they did not report on the Central American region. The rate of vancomycin-resistant *E. faecalis* was low (1.6%), and this was consistent year by year. This rate was similar to that reported by Sader et al. (2.3%) [18], and similar the global rate for the 2004–2013 T.E.S.T. study period (2.2%) [4]. There was a striking regional pattern among *E. faecalis* isolates: none of the *E. faecalis* isolates collected in Central America as part of this study were vancomycin-resistant. Importantly, the high rates of susceptibility of these *Enterococcus* spp. to linezolid and tigecycline were maintained among vancomycin-resistant isolates. Indeed, Sader et al. [18] reported 100% susceptibility of *Enterococcus* spp. to tigecycline. Three *E. faecium* isolates collected in this T.E.S.T. study were non-susceptible to tigecycline, all of which were vancomycin-resistant (two collected in 2008 and one in 2012). All vancomycin-resistant *E. faecalis* isolates were susceptible to tigecycline.

High frequencies of ESBL-producing Enterobacteriaceae have been reported in Latin America by previous surveillance studies, particularly *K. pneumoniae* and *E. coli* [2]. In this update we have shown the rate of these organisms to be 36.3% and 25.4%. Sader et al. [18] reported higher rates of ESBL-producing *K. pneumoniae* and *E. coli* (57.3 and 37.7%, respectively) from the SENTRY study of Latin American centers (2011–2014). Differences could be in part due to the different countries included in each study, and variable rates of ESBL production across Latin America have previously been reported [2]. Furthermore, the rates of ESBL production in this T.E.S.T. study have been shown to vary widely by country. Year on year the rates of ESBL production were relatively stable which supports the findings of Kazmierczak et al. [22] for *K. pneumoniae* collected from intra-abdominal infections in Latin America between 2008 and 2012. We found a high percentage of resistance to fluoroquinolones (levofloxacin) among *E. coli* isolates (48.8%), reflecting the wide use of this antimicrobial in the treatment of urinary tract infections in Latin America. The resistance rate among ESBL-producing isolates of *E. coli* was higher (84.4%).

Carbapenem-resistant Enterobacteriaceae are of particular concern as they are increasingly reported globally and few treatment options are available for these types of infections [23, 24]. In this study, 3.8% (482/12,839) of Enterobacteriaceae were meropenem-resistant. This rate is the same as the Latin American rate of meropenem resistance reported by Sader et al. [18] for isolates collected between 2011 and 2014 (3.8%). The majority of meropenem-resistant Enterobacteriaceae in this T.E.S.T. study were *K. pneumoniae* isolates [54.1% (261/482)]. Carbapenem-resistant *K. pneumoniae* are often co-resistant with fluoroquinolones, tetracycline derivatives and aminoglycosides, and in this study approximately 50% of such isolates were non-susceptible to amikacin and/or minocycline and 90% were resistant to levofloxacin (data not shown). The World Health Organization performed a review of published studies (1946–2013) and reported that for patients with carbapenem-resistant *K. pneumoniae* infections there was a significant increase in all-cause mortality and 30-day mortality [25]. The agent most active against the carbapenem-resistant *K. pneumoniae* isolates in this study was tigecycline (87.3%, 233/267), followed by amikacin and minocycline [50.9% (136/267) and 48.3% (129/267) respectively].

*Acinetobacter baumannii* and *P. aeruginosa* are clinically important pathogens and major causes of healthcare-associated infections [26, 27]. These pathogens are difficult to treat because, in addition to their intrinsic resistance to many antimicrobials, they have the ability to acquire resistance by a range of mechanisms [26]. In this study, 70.3% of the *A. baumannii* isolates and 26.7% of the *P. aeruginosa* isolates submitted between 2004 and 2015 were MDR. A study of T.E.S.T. data for 2004–2014 reported a global rate of MDR *A. baumannii* of 44.3% and Latin America had one of highest regional rates (Latin America, 70.5%; Middle East, 69.5%; Africa, 61.2%) [28]. Global rates of MDR *A. baumannii* isolates increased over the 2004–2014 time period, however the results of this study show rates of MDR in Latin American were variable between 2004 and 2015. Indeed, *A. baumannii* MDR rates decreased each year from 2011 to 2015. Among *A. baumannii* isolates, tigecycline had the lowest MIC_90_ (2 mg/L) of the antimicrobials on the T.E.S.T. panel. This MIC_90_ was comparable with the SENTRY study which reported an MIC_90_ of 2 mg/L for *Acinetobacter* spp. collected in Latin America between 2011 and 2014 [18], and lower than the MIC_90_ reported by Jones et al. [1] for *Acinetobacter* spp. collected in Latin America in 2011 (MIC_90_ 4 mg/L). The antimicrobial with the highest rate of susceptibility against *A. baumannii* collected in this study was minocycline (88.3%). Susceptibility to meropenem was 25.7%, which is lower than the Latin American rate reported from the T.E.S.T. study for the 2004–2010 time period (33.9%) [10], and lower than the global rate for the 2004–2013 T.E.S.T. study period (54.8%) [4]. The year on year data from this study between 2006 and 2015 shows a trend of decreasing *A. baumannii* susceptibility to meropenem. *Acinetobacter* spp. strains resistant to carbapenems have increased in prevalence and present a serious treatment challenge to clinicians [27]. As a result older agents, such as colistin, have seen a resurgence in use; however, colistin-resistant and pan-drug-resistant strains have been reported [8, 27, 29] highlighting the importance of judicious antimicrobial use and stewardship.

It is notable, particularly in the case of the Enterobacteriaceae, that from the start of this study until 2009/2010 susceptibility to minocycline decreased and then from 2010/2011 onwards began to increase again so that rates in 2015 are similar to rates from 2004. This has also been reported in both a global analysis of the T.E.S.T. data and also among isolates from skin and soft tissue infections [4, 30]. The reasons for this are unclear although there was variability in center involvement throughout the study and the total number of isolates submitted peaked in 2009 with lower numbers of isolates submitted in subsequent years. To our knowledge this has not be reported by other surveillance studies and warrants further analysis.

Surveillance studies such as T.E.S.T are an invaluable tool for monitoring the rate of resistant pathogen phenotypes and antimicrobial susceptibility among clinical pathogens. However, there are a number of limitations to this study. For example, there was a yearly variation in the number of participating centers with a larger number of centers participating in the earlier years of the study than the latter. The center count was at its highest in 2008 (44 centers) and at its lowest in 2012 (4 centers). Furthermore, the number of isolates submitted varied widely from country to country, with almost half of isolates (48.9%) being submitted by Mexico and Argentina combined.

## Conclusions

Antimicrobial resistance continues to be a problem in Latin America with high rates of MRSA, ESBL-producing Enterobacteriaceae and MDR *A. baumannii*. There are limited treatment choices for infections caused by such organisms; however, this study shows that linezolid, vancomycin and tigecycline continue to be active in vitro against Gram-positive organisms such as MRSA. Against resistant Gram-negative organisms, both in Latin America and globally, the rise in antimicrobial resistance is more troubling especially in the context of carbapenem resistance. In vitro, this study reported high percentages of susceptibility to meropenem and tigecycline among Gram-negative organisms (with the exception of *P. aeruginosa*). However, resistant isolates were identified and warrant continued monitoring.

## Additional file

**Additional file 1: Table S1.** Antimicrobial susceptibility rates among Gram-positive organisms collected in Latin America by year, 2004–2015. **Table S2.** Antimicrobial susceptibility rates among Gram-negative organisms collected in Latin America by year, 2004–2015.

## Abbreviations

CLSI: Clinical and Laboratory Standards Institute
ESBL: extended-spectrum β-lactamase
FDA: US Food and Drugs Administration
MIC: minimum inhibitory concentration
MRSA: methicillin-resistant *staphylococcus aureus*
MDR: multidrug-resistant
T.E.S.T.: Tigecycline Evaluation and Surveillance Trial
