# Supplementary material for: Antimicrobial susceptibility among Gram-positive and Gram-negative organisms collected from the Latin American region between 2004 and 2015 as part of the Tigecycline Evaluation and Surveillance Trial
Source: Ann Clin Microbiol Antimicrob. 2017 Jul 12;16:50. doi: 10.1186/s12941-017-0222-0 (PMC5508790; doi:10.1186/s12941-017-0222-0)
Supplement: Supplementary file 1 — Additional file 1: Table S1. Antimicrobial susceptibility rates among Gram-positive organisms collected in Latin America by year, 2004–2015. Table S2. Antimicrobial susceptibility rates among Gram-negative organisms collected in Latin America by year, 2004–2015. [file 12941_2017_222_MOESM1_ESM.docx]

**Table S1.** Antimicrobial susceptibility rates among Gram-positive organisms collected in Latin America by year, 2004–2015.

| **Species and antimicrobial agent** | **% Susceptible** | | | | | | | | | | | |
| --- | --- | --- | --- | --- | --- | --- | --- | --- | --- | --- | --- | --- |
|  | **2004** | **2005** | **2006** | **2007** | **2008** | **2009** | **2010** | **2011** | **2012** | **2013** | **2014** | **2015** |
| ***S. aureus*** | **N = 66** | **N = 266** | **N = 512** | **N = 535** | **N = 821** | **N = 924** | **N = 625** | **N = 108** | **N = 99** | **N = 145** | **N = 111** | **N = 351** |
| Amox-clav | - | - | - | - | - | - | - | - | - | - | - | - |
| Ampicillin | - | - | - | - | - | - | - | - | - | - | - | - |
| Ceftriaxone | - | - | - | - | - | - | - | - | - | - | - | - |
| Levofloxacin | 56.1 | 54.5 | 55.5 | 57.6 | 60.5 | 53.8 | 52.6 | 61.1 | 64.6 | 69.0 | 58.6 | 74.9 |
| Linezolid | 100 | 100 | 100 | 100 | 100 | 100 | 100 | 100 | 100 | 100 | 100 | 100 |
| Meropenem | - | - | - | - | - | - | - | - | - | - | - | - |
| Minocycline | 98.5 | 99.2 | 99.0 | 99.1 | 96.1 | 98.3 | 93.3 | 99.1 | 100 | 100 | 100 | 98.0 |
| Penicillin | 10.6 | 4.1 | 6.1 | 6.8 | 4.4 | 4.9 | 5.4 | 2.8 | 6.1 | 7.6 | 2.7 | 8.5 |
| Pip-taz | - | - | - | - | - | - | - | - | - | - | - | - |
| Tigecycline | 100 | 100 | 100 | 100 | 100 | 99.7 | 100 | 100 | 100 | 100 | 100 | 100 |
| Vancomycin | 100 | 100 | 100 | 100 | 100 | 100 | 100 | 100 | 100 | 100 | 100 | 100 |
| ***S. aureus*, MR** | **N = 30** | **N = 131** | **N = 247** | **N = 258** | **N = 374** | **N = 479** | **N = 325** | **N = 57** | **N = 47** | **N = 55** | **N = 60** | **N = 139** |
| Levofloxacin | 3.3 | 10.7 | 15.0 | 16.3 | 21.7 | 17.7 | 19.1 | 29.8 | 27.7 | 27.3 | 26.7 | 46.8 |
| Linezolid | 100 | 100 | 100 | 100 | 100 | 100 | 100 | 100 | 100 | 100 | 100 | 100 |
| Minocycline | 96.7 | 99.2 | 98.4 | 98.4 | 92.5 | 98.1 | 90.5 | 100 | 100 | 100 | 100 | 95.7 |
| Tigecycline | 100 | 100 | 100 | 100 | 100 | 99.4 | 100 | 100 | 100 | 100 | 100 | 100 |
| Vancomycin | 100 | 100 | 100 | 100 | 100 | 100 | 100 | 100 | 100 | 100 | 100 | 100 |
| ***S. pneumoniae*** | **N = 41** | **N = 123** | **N = 178** | **N = 232** | **N = 269** | **N = 258** | **N = 91** | **N = 52** | **N = 14** | **N = 49** | **N = 44** | **N = 85** |
| Amox-clav | 100 | 98.4 | 96.6 | 97.0 | 94.1 | 92.6 | 91.2 | 94.2 | 100 | 87.8 | 84.1 | 96.5 |
| Ampicillin | - | - | - | - | - | - | - | - | - | - | - | - |
| Azithromycin | 77.4 (31) | 81.3 (96) | 68.0 (147) | 71.3 (167) | 76.1 (247) | 71.0 (245) | 71.3 (87) | 79.6 (49) | 92.3 (13) | 61.1 (36) | 61.4 | 70.6 |
| Ceftriaxone | 100 | 98.4 | 94.9 | 99.1 | 95.5 | 89.1 | 94.5 | 90.4 | 100 | 93.9 | 93.2 | 95.3 |
| Clarithromycin | 77.4 (31) | 81.3 (96) | 68.0 (147) | 71.9 (167) | 76.5 (247) | 71.4 (245) | 70.1 (87) | 79.6 (49) | 84.6 (13) | 61.1 (36) | 61.4 | 70.6 |
| Clindamycin | 87.1 (31) | 94.8 (96) | 84.4 (147) | 91.0 (167) | 87.0 (247) | 91.4 (245) | 88.5 (87) | 89.8 (49) | 92.3 (13) | 75.0 (36) | 75.0 | 83.5 |
| Erythromycin | 77.4 (31) | 80.2 (96) | 67.3 (147) | 71.9 (167) | 76.9 (247) | 71.0 (245) | 58.6 (87) | 79.6 (49) | 92.3 (13) | 61.1 (36) | 61.4 | 69.4 |
| Levofloxacin | 100 | 99.2 | 99.4 | 98.7 | 99.6 | 97.7 | 98.9 | 100 | 100 | 98.0 | 100 | 97.6 |
| Linezolid | 100 | 100 | 100 | 100 | 100 | 100 | 100 | 100 | 100 | 100 | 100 | 100 |
| Meropenem | 85.7 (21) | 84.2 (38) | 76.0 (104) | 82.7 (231) | 74.7 | 79.1 | 80.2 | 88.5 | 100 | 79.6 | 65.9 | 87.1 |
| Minocycline | 92.7 | 82.9 | 79.2 | 75.0 | 46.1 | 32.2 | 33.0 | 59.6 | 85.7 | 65.3 | 70.5 | 71.8 |
| Penicillin | 68.3 | 68.3 | 59.6 | 56.0 | 50.6 | 46.1 | 48.4 | 67.3 | 85.7 | 44.9 | 54.5 | 54.1 |
| Pip-taz | - | - | - | - | - | - | - | - | - | - | - | - |
| Tigecycline | 78.0 | 84.6 | 90.4 | 95.7 | 97.4 | 99.2 | 100 | 100 | 100 | 100 | 100 | 100 |
| Vancomycin | 100 | 100 | 100 | 100 | 100 | 100 | 100 | 100 | 100 | 100 | 100 | 100 |
| ***S. pneumoniae*, PR** | **N = 2** | **N = 10** | **N = 19** | **N = 30** | **N = 49** | **N = 43** | **N = 14** | **N = 6** | **N = 0** | **N = 8** | **N = 9** | **N = 8** |
| Amox-clav | [2] | 80.0 | 68.4 | 76.7 | 67.3 | 55.8 | 57.1 | [4] | –– | [2] | [4] | [5] |
| Ampicillin | - | - | - | - | - | - | - | - | - | - | - | - |
| Azithromycin | [2] | [5] (9) | 47.1 (17) | 47.4 (19) | 35.4 (48) | 45.2 (42) | 53.8 (13) | [3] | –– | [1] (6) | [0] | [3] |
| Ceftriaxone | [2] | 80.0 | 57.9 | 93.3 | 81.6 | 44.2 | 71.4 | [1] | –– | [5] | [7] | [6] |
| Clarithromycin | [2] | [5] (9) | 47.1 (17) | 47.4 (19) | 35.4 (48) | 45.2 (42) | 53.8 (13) | [3] | –– | [1] (6) | [0] | [3] |
| Clindamycin | [2] | [8] (9) | 58.8 (17) | 78.9 (19) | 60.4 (48) | 66.7 (42) | 69.2 (13) | [4] | –– | [1] (6) | [1] | [4] |
| Erythromycin | [2] | [5] (9) | 47.1 (17) | 47.4 (19) | 35.4 (48) | 45.2 (42) | 53.8 (13) | [3] | –– | [1] (6) | [0] | [3] |
| Levofloxacin | [2] | 90.0 | 100 | 100 | 100 | 93.0 | 100 | [6] | –– | [8] | [9] | [8] |
| Linezolid | [2] | 100 | 100 | 100 | 100 | 100 | 100 | [6] | –– | [8] | [9] | [8] |
| Meropenem | [0] | [0] (4) | 0.0 (11) | 10.0 | 6.1 | 9.3 | 28.6 | [0] | –– | [0] | [0] | [0] |
| Minocycline | [2] | 70.0 | 47.4 | 46.7 | 26.5 | 14.0 | 21.4 | [1] | –– | [2] | [4] | [4] |
| Pip-taz | - | - | - | - | - | - | - | - | - | - | - | - |
| Tigecycline | [2] | 90.0 | 100 | 93.3 | 98.0 | 100 | 100 | [6] | –– | [8] | [9] | [8] |
| Vancomycin | [2] | 100 | 100 | 100 | 100 | 100 | 100 | [6] | –– | [8] | [9] | [8] |
| ***S. agalactiae*** | **N = 14** | **N = 98** | **N = 161** | **N = 199** | **N = 258** | **N = 235** | **N = 112** | **N = 45** | **N = 21** | **N = 47** | **N = 44** | **N = 105** |
| Amox-clav | - | - | - | - | - | - | - | - | - | - | - | - |
| Ampicillin | 100 | 100 | 100 | 100 | 100 | 100 | 100 | 100 | 100 | 100 | 100 | 100 |
| Ceftriaxone | 100 | 100 | 100 | 99.5 | 100 | 99.1 | 100 | 100 | 100 | 100 | 100 | 100 |
| Levofloxacin | 100 | 99.0 | 99.4 | 99.5 | 97.7 | 99.1 | 94.6 | 100 | 95.2 | 100 | 77.3 | 97.1 |
| Linezolid | 100 | 100 | 100 | 100 | 100 | 100 | 100 | 100 | 100 | 100 | 100 | 100 |
| Meropenem | –– | 100 (28) | 100 (104) | 100 | 100 | 100 | 100 | 100 | 100 | 100 | 100 | 100 |
| Minocycline | 21.4 | 49.0 | 26.1 | 21.6 | 32.2 | 31.1 | 15.2 | 6.7 | 28.6 | 14.9 | 27.3 | 31.4 |
| Penicillin | 100 | 100 | 100 | 100 | 100 | 100 | 100 | 100 | 100 | 100 | 100 | 100 |
| Pip-taz | - | - | - | - | - | - | - | - | - | - | - | - |
| Tigecycline | 100 | 100 | 99.4 | 99.5 | 100 | 100 | 100 | 100 | 100 | 100 | 100 | 100 |
| Vancomycin | 100 | 100 | 100 | 100 | 100 | 100 | 100 | 100 | 100 | 100 | 100 | 100 |
| ***E. faecium*** | **N = 13** | **N = 12** | **N = 65** | **N = 55** | **N = 117** | **N = 138** | **N = 78** | **N = 8** | **N = 12** | **N = 26** | **N = 13** | **N = 39** |
| Amox-clav | - | - | - | - | - | - | - | - | - | - | - | - |
| Ampicillin | 38.5 | 25.0 | 24.6 | 18.2 | 22.2 | 34.8 | 32.1 | [4] | 25.0 | 11.5 | 23.1 | 10.3 |
| Ceftriaxone | - | - | - | - | - | - | - | - | - | - | - | - |
| Levofloxacin | 30.8 | 16.7 | 16.9 | 21.8 | 23.9 | 23.9 | 17.9 | [3] | 33.3 | 15.4 | 23.1 | 17.9 |
| Linezolid | 100 | 100 | 100 | 100 | 100 | 99.3 | 100 | [8] | 100 | 100 | 100 | 100 |
| Meropenem | - | - | - | - | - | - | - | - | - | - | - | - |
| Minocycline | 84.6 | 58.3 | 58.5 | 65.5 | 53.8 | 73.9 | 50.0 | [4] | 66.7 | 46.2 | 69.2 | 71.8 |
| Penicillin | 38.5 | 25.0 | 23.1 | 10.9 | 19.7 | 32.6 | 24.4 | [4] | 25.0 | 3.8 | 15.4 | 10.3 |
| Pip-taz | - | - | - | - | - | - | - | - | - | - | - | - |
| Tigecycline | 100 | 100 | 100 | 100 | 98.3 | 100 | 100 | [8] | 91.7 | 100 | 100 | 100 |
| Vancomycin | 38.5 | 25.0 | 60.0 | 52.7 | 47.0 | 68.8 | 64.1 | [6] | 58.3 | 38.5 | 46.2 | 56.4 |
| ***E. faecium*, VR** | **N = 8** | **N = 9** | **N = 24** | **N = 22** | **N = 60** | **N = 40** | **N = 25** | **N = 2** | **N = 5** | **N = 16** | **N = 7** | **N = 17** |
| Amox-clav | - | - | - | - | - | - | - | - | - | - | - | - |
| Ampicillin | [1] | [1] | 0.0 | 0.0 | 0.0 | 0.0 | 0.0 | [0] | [0] | 0.0 | [0] | 0.0 |
| Ceftriaxone | - | - | - | - | - | - | - | - | - | - | - | - |
| Levofloxacin | [0] | [0] | 0.0 | 4.5 | 1.7 | 0.0 | 0.0 | [0] | [0] | 0.0 | [0] | 0.0 |
| Linezolid | [8] | [9] | 100 | 100 | 100 | 100 | 100 | [2] | [5] | 100 | [7] | 100 |
| Meropenem | - | - | - | - | - | - | - | - | - | - | - | - |
| Minocycline | [7] | [5] | 87.5 | 77.3 | 60.0 | 85.0 | 56.0 | [1] | [3] | 56.3 | [6] | 88.2 |
| Penicillin | [1] | [1] | 0.0 | 0.0 | 1.7 | 0.0 | 0.0 | [0] | [0] | 0.0 | [0] | 0.0 |
| Pip-taz | - | - | - | - | - | - | - | - | - | - | - | - |
| Tigecycline | [8] | [9] | 100 | 100 | 96.7 | 100 | 100 | [2] | [4] | 100 | [7] | 100 |
| ***E. faecalis*** | **N = 25** | **N = 104** | **N = 231** | **N = 216** | **N = 404** | **N = 389** | **N = 258** | **N = 40** | **N = 46** | **N = 71** | **N = 56** | **N = 164** |
| Amox-clav | - | - | - | - | - | - | - | - | - | - | - | - |
| Ampicillin | 100 | 100 | 100 | 100 | 99.0 | 99.7 | 98.4 | 97.5 | 89.1 | 98.6 | 100 | 97.6 |
| Ceftriaxone | - | - | - | - | - | - | - | - | - | - | - | - |
| Levofloxacin | 88.0 | 71.2 | 73.6 | 66.2 | 67.1 | 69.7 | 58.9 | 90.0 | 60.9 | 77.5 | 75.0 | 76.8 |
| Linezolid | 100 | 100 | 100 | 100 | 100 | 99.2 | 99.6 | 100 | 100 | 100 | 100 | 100 |
| Meropenem | - | - | - | - | - | - | - | - | - | - | - | - |
| Minocycline | 56.0 | 45.2 | 38.1 | 35.2 | 34.2 | 35.2 | 27.9 | 20.0 | 32.6 | 31.0 | 41.1 | 36.0 |
| Penicillin | 100 | 98.1 | 99.6 | 97.7 | 99.0 | 99.2 | 97.7 | 95.0 | 89.1 | 97.2 | 100 | 98.2 |
| Pip-taz | - | - | - | - | - | - | - | - | - | - | - | - |
| Tigecycline | 100 | 100 | 98.7 | 100 | 100 | 99.7 | 99.2 | 100 | 100 | 100 | 100 | 100 |
| Vancomycin | 100 | 91.3 | 97.8 | 96.8 | 99.5 | 97.9 | 100 | 100 | 95.7 | 100 | 100 | 96.3 |
| ***E. faecalis*, VR** | **N = 0** | **N = 7** | **N = 4** | **N = 6** | **N = 2** | **N = 7** | **N = 0** | **N = 0** | **N = 2** | **N = 0** | **N = 0** | **N = 5** |
| Amox-clav | - | - | - | - | - | - | - | - | - | - | - | - |
| Ampicillin | –– | [7] | [4] | [6] | [1] | [6] | –– | –– | [0] | –– | –– | [2] |
| Ceftriaxone | - | - | - | - | - | - | - | - | - | - | - | - |
| Levofloxacin | –– | [0] | [0] | [0] | [0] | [1] | –– | –– | [0] | –– | –– | [1] |
| Linezolid | –– | [7] | [4] | [6] | [2] | [7] | –– | –– | [2] | –– | –– | [5] |
| Meropenem | - | - | - | - | - | - | - | - | - | - | - | - |
| Minocycline | –– | [5] | [3] | [3] | [1] | [3] | –– | –– | [0] | –– | –– | [2] |
| Penicillin | –– | [5] | [4] | [6] | [1] | [6] | –– | –– | [0] | –– | –– | [3] |
| Pip-taz | - | - | - | - | - | - | - | - | - | - | - | - |
| Tigecycline | –– | [7] | [4] | [6] | [2] | [7] | –– | –– | [2] | –– | –– | [5] |

‘––‘ No isolates were tested against this agent.

‘-‘ no CLSI breakpoints available.

When N<10 percentages are not given, instead the total number of susceptible isolates are shown in square brackets.

When fewer than the total number of isolates collected were tested against a given antimicrobial, the number of isolates tested are shown in parentheses after the percentage susceptibility value.

Amox-clav, amoxicillin-clavulanic acid; MR, methicillin-resistant; PR, penicillin-resistant; pip-taz, piperacillin-tazobactam; VR, vancomycin-resistant.

**Table S2.** Antimicrobial susceptibility rates among Gram-negative organisms collected in Latin America by year, 2004–2015.

| **Species and antimicrobial agent** | **% Susceptible** | | | | | | | | | | | |
| --- | --- | --- | --- | --- | --- | --- | --- | --- | --- | --- | --- | --- |
|  | **2004** | **2005** | **2006** | **2007** | **2008** | **2009** | **2010** | **2011** | **2012** | **2013** | **2014** | **2015** |
| ***K. pneumoniae*** | **N = 67** | **N = 202** | **N = 444** | **N = 462** | **N = 791** | **N = 851** | **N = 525** | **N = 87** | **N = 74** | **N = 117** | **N = 91** | **N = 321** |
| Amikacin | 95.5 | 85.6 | 88.5 | 82.7 | 82.0 | 85.1 | 91.0 | 89.7 | 97.3 | 91.5 | 96.7 | 92.5 |
| Amox-clav | 53.7 | 43.6 | 50.0 | 46.5 | 47.0 | 48.6 | 44.8 | 46.0 | 60.8 | 41.0 | 45.1 | 47.7 |
| Ampicillin | 1.5 | 1.5 (200) | 2.3 (439) | 2.2 (461) | 0.3 | 1.8 | 0.6 | 1.1 | 0.0 | 0.0 | 2.2 | 1.6 |
| Cefepime | 65.7 | 50.5 | 56.1 | 54.1 | 57.9 | 55.9 | 55.6 | 66.7 | 68.9 | 49.6 | 50.5 | 51.1 |
| Ceftriaxone | 64.2 | 46.5 | 47.1 | 49.8 | 49.8 | 48.9 | 47.4 | 62.1 | 58.1 | 45.3 | 46.2 | 47.7 |
| Levofloxacin | 83.6 | 58.9 | 61.7 | 69.9 | 65.0 | 64.6 | 61.0 | 78.2 | 83.8 | 64.1 | 68.1 | 65.1 |
| Meropenem | [2] (2) | 100 (14) | 88.3 (222) | 95.7 (460) | 92.9 | 89.2 | 91.8 | 93.1 | 93.2 | 86.3 | 89.0 | 85.0 |
| Minocycline | 80.6 | 75.7 | 72.3 | 68.2 | 54.4 | 52.4 | 44.4 | 50.6 | 78.4 | 72.6 | 82.4 | 81.9 |
| Pip-taz | 67.2 | 58.4 | 69.1 | 66.9 | 59.5 | 62.7 | 58.9 | 66.7 | 82.4 | 64.1 | 73.6 | 71.0 |
| Tigecycline | 97.0 | 96.5 | 95.3 | 97.6 | 94.7 | 95.8 | 95.0 | 98.9 | 93.2 | 91.5 | 97.8 | 96.3 |
| ***K. pneumoniae*, ESBL** | **N = 23** | **N = 101** | **N = 166** | **N = 196** | **N = 263** | **N = 299** | **N = 187** | **N = 29** | **N = 22** | **N = 38** | **N = 43** | **N = 98** |
| Amikacin | 87.0 | 77.2 | 76.5 | 66.3 | 62.7 | 73.9 | 81.8 | 75.9 | 90.9 | 92.1 | 97.7 | 91.8 |
| Amox-clav | 0.0 | 5.9 | 20.5 | 10.2 | 10.6 | 18.1 | 10.7 | 3.4 | 18.2 | 10.5 | 11.6 | 11.2 |
| Ampicillin | 0.0 | 0.0 | 0.0 | 0.5 | 0.0 | 0.0 | 0.0 | 0.0 | 0.0 | 0.0 | 0.0 | 0.0 |
| Cefepime | 4.3 | 8.9 | 11.4 | 7.7 | 17.1 | 11.4 | 11.8 | 10.3 | 27.3 | 5.3 | 7.0 | 5.1 |
| Ceftriaxone | 0.0 | 2.0 | 0.6 | 2.0 | 0.4 | 0.7 | 1.1 | 0.0 | 0.0 | 0.0 | 2.3 | 1.0 |
| Levofloxacin | 56.5 | 30.7 | 34.9 | 44.9 | 37.6 | 40.1 | 34.8 | 48.3 | 63.6 | 50.0 | 34.9 | 41.8 |
| Meropenem | [2] (2) | [3] (3) | 85.6 (90) | 93.9 | 87.8 | 85.6 | 85.0 | 86.2 | 81.8 | 86.8 | 81.4 | 81.6 |
| Minocycline | 69.6 | 70.3 | 65.1 | 57.1 | 43.3 | 37.1 | 25.7 | 44.8 | 72.7 | 71.1 | 86.0 | 84.7 |
| Pip-taz | 21.7 | 27.7 | 41.0 | 36.2 | 27.8 | 38.8 | 28.3 | 20.7 | 50.0 | 47.4 | 53.5 | 49.0 |
| Tigecycline | 91.3 | 95.0 | 93.4 | 95.4 | 92.0 | 93.3 | 92.5 | 96.6 | 81.8 | 84.2 | 97.7 | 98.0 |
| ***K. oxytoca*** | **N = 16** | **N = 19** | **N = 64** | **N = 44** | **N = 69** | **N = 83** | **N = 57** | **N = 8** | **N = 7** | **N = 17** | **N = 11** | **N = 14** |
| Amikacin | 93.8 | 100 | 95.3 | 95.5 | 92.8 | 94.0 | 98.2 | [8] | [7] | 94.1 | 100 | 100 |
| Amox-clav | 87.5 | 68.4 | 76.6 | 68.2 | 72.5 | 65.1 | 61.4 | [4] | [6] | 88.2 | 54.5 | 85.7 |
| Ampicillin | 0.0 | 0.0 | 0.0 (61) | 2.3 | 1.4 | 0.0 | 5.3 | [0] | [0] | 11.8 | 0.0 | 0.0 |
| Cefepime | 81.3 | 73.7 | 81.3 | 84.1 | 76.8 | 73.5 | 80.7 | [7] | [5] | 94.1 | 72.7 | 92.9 |
| Ceftriaxone | 81.3 | 63.2 | 70.3 | 75.0 | 71.0 | 65.1 | 61.4 | [5] | [5] | 94.1 | 45.5 | 92.9 |
| Levofloxacin | 100 | 89.5 | 81.3 | 90.9 | 85.5 | 71.1 | 71.9 | [7] | [6] | 100 | 100 | 100 |
| Meropenem | –– | –– | 100 (24) | 100 | 97.1 (68) | 95.2 | 100 | [8] | [7] | 94.1 | 100 | 92.9 |
| Minocycline | 100 | 84.2 | 79.7 | 84.1 | 79.7 | 72.3 | 68.4 | [5] | [7] | 82.4 | 100 | 85.7 |
| Pip-taz | 93.8 | 84.2 | 92.2 | 81.8 | 81.2 | 83.1 | 77.2 | [6] | [7] | 100 | 72.7 | 92.9 |
| Tigecycline | 100 | 94.7 | 93.8 | 100 | 100 | 97.6 | 100 | [7] | [7] | 100 | 100 | 100 |
| ***K. oxytoca*, ESBL** | **N = 2** | **N = 8** | **N = 10** | **N = 6** | **N = 8** | **N = 13** | **N = 14** | **N = 0** | **N = 1** | **N = 0** | **N = 4** | **N = 1** |
| Amikacin | [1] | [8] | 80.0 | [6] | [6] | 84.6 | 100 | — | [1] | — | [4] | [1] |
| Amox-clav | [1] | [2] | 40.0 | [0] | [4] | 23.1 | 21.4 | — | [0] | — | [1] | [0] |
| Ampicillin | [0] | [0] | 0.0 | [0] | [0] | 0.0 | 0.0 | — | [0] | — | [0] | [0] |
| Cefepime | [0] | [3] | 10.0 | [0] | [1] | 23.1 | 64.3 | — | [0] | — | [1] | [0] |
| Ceftriaxone | [0] | [1] | 0.0 | [0] | [0] | 0.0 | 7.1 | — | [0] | — | [0] | [0] |
| Levofloxacin | [2] | [7] | 30.0 | [5] | [4] | 38.5 | 35.7 | — | [1] | — | [4] | [1] |
| Meropenem | –– | –– | [2] (2) | [6] | [7] (7) | 92.3 | 100 | — | [1] | — | [4] | [0] |
| Minocycline | [2] | [5] | 20.0 | [3] | [5] | 38.5 | 42.9 | — | [1] | — | [4] | [0] |
| Pip-taz | [2] | [5] | 80.0 | [1] | [4] | 69.2 | 57.1 | — | [1] | — | [3] | [0] |
| Tigecycline | [2] | [7] | 70.0 | [6] | [8] | 100 | 100 | — | [1] | — | [4] | [1] |
| ***E. coli*** | **N = 73** | **N = 211** | **N = 588** | **N = 534** | **N = 893** | **N = 1050** | **N = 660** | **N = 125** | **N = 102** | **N = 151** | **N = 105** | **N = 420** |
| Amikacin | 98.6 | 98.1 | 96.8 | 95.9 | 92.6 | 95.0 | 96.2 | 95.2 | 98.0 | 98.7 | 96.2 | 98.8 |
| Amox-clav | 67.1 | 67.3 | 52.6 | 51.3 | 48.5 | 49.1 | 47.1 | 57.6 | 51.0 | 55.6 | 54.3 | 56.9 |
| Ampicillin | 37.0 | 32.7 | 21.8 | 23.8 | 23.0 | 18.6 | 15.9 | 20.8 | 23.5 | 17.9 | 21.0 | 21.0 |
| Cefepime | 90.4 | 82.9 | 68.2 | 65.5 | 67.9 | 68.0 | 67.7 | 64.0 | 67.6 | 65.6 | 60.0 | 60.0 |
| Ceftriaxone | 90.4 | 77.7 | 60.9 | 59.4 | 62.5 | 60.6 | 56.7 | 58.4 | 51.0 | 52.3 | 49.5 | 54.3 |
| Levofloxacin | 78.1 | 68.2 | 47.1 | 52.6 | 46.2 | 45.7 | 42.0 | 50.4 | 40.2 | 38.4 | 45.7 | 50.0 |
| Meropenem | [1] (1) | [1] (1) | 95.8 (264) | 97.7 (529) | 97.5 (876) | 98.2 | 99.5 | 100 | 96.1 | 100 | 94.3 | 99.0 |
| Minocycline | 76.7 | 74.4 | 66.3 | 68.4 | 54.8 | 56.1 | 49.8 | 48.0 | 74.5 | 66.9 | 81.9 | 81.7 |
| Pip-taz | 91.8 | 93.8 | 90.3 | 86.3 | 81.4 | 83.3 | 82.7 | 80.8 | 95.1 | 92.1 | 87.6 | 89.0 |
| Tigecycline | 100 | 100 | 100 | 99.6 | 99.6 | 99.7 | 99.8 | 99.2 | 99.0 | 100 | 100 | 99.8 |
| ***E. coli*, ESBL** | **N = 6** | **N = 37** | **N = 164** | **N = 147** | **N = 220** | **N = 272** | **N = 158** | **N = 30** | **N = 15** | **N = 45** | **N = 37** | **N = 115** |
| Amikacin | [5] | 91.9 | 92.7 | 90.5 | 83.2 | 90.1 | 93.0 | 93.3 | 93.3 | 95.6 | 94.6 | 95.7 |
| Amox-clav | [0] | 24.3 | 28.0 | 21.1 | 19.1 | 22.4 | 16.5 | 23.3 | 20.0 | 35.6 | 35.1 | 33.0 |
| Ampicillin | [0] | 0.0 | 0.0 | 0.7 | 1.8 | 1.1 | 0.0 | 0.0 | 0.0 | 0.0 | 2.7 | 0.0 |
| Cefepime | [0] | 10.8 | 9.8 | 9.5 | 12.7 | 13.2 | 12.0 | 6.7 | 26.7 | 8.9 | 13.5 | 4.3 |
| Ceftriaxone | [0] | 0.0 | 0.0 | 0.7 | 2.7 | 2.6 | 0.6 | 0.0 | 0.0 | 0.0 | 0.0 | 0.9 |
| Levofloxacin | [2] | 24.3 | 6.7 | 17.0 | 12.7 | 10.7 | 10.1 | 3.3 | 6.7 | 8.9 | 10.8 | 14.8 |
| Meropenem | –– | –– | 98.7 (77) | 97.9 (143) | 94.8 (211) | 96.0 | 100 | 100 | 93.3 | 100 | 91.9 | 97.4 |
| Minocycline | [4] | 64.9 | 62.2 | 60.5 | 43.2 | 51.8 | 38.0 | 53.3 | 66.7 | 64.4 | 73.0 | 80.9 |
| Pip-taz | [4] | 81.1 | 88.4 | 74.8 | 64.1 | 72.8 | 65.2 | 46.7 | 93.3 | 91.1 | 86.5 | 82.6 |
| Tigecycline | [6] | 100 | 100 | 99.3 | 100 | 99.6 | 100 | 100 | 100 | 100 | 100 | 100 |
| ***Enterobacter* spp.** | **N = 58** | **N = 198** | **N = 432** | **N = 491** | **N = 712** | **N = 745** | **N = 504** | **N = 94** | **N = 69** | **N = 123** | **N = 100** | **N = 292** |
| Amikacin | 98.3 | 87.4 | 88.4 | 87.6 | 88.5 | 88.2 | 93.8 | 77.7 | 94.2 | 96.7 | 95.0 | 98.6 |
| Amox-clav | 8.6 | 3.0 | 5.1 | 5.1 | 5.3 | 5.5 | 6.3 | 7.4 | 2.9 | 1.6 | 2.0 | 4.1 |
| Ampicillin | 5.2 | 1.5 | 0.9 (426) | 2.0 (489) | 6.2 | 3.4 | 2.6 | 3.2 | 7.2 | 4.1 | 8.0 | 6.2 |
| Cefepime | 82.8 | 61.1 | 68.3 | 68.8 | 68.8 | 66.6 | 76.6 | 78.7 | 75.4 | 78.9 | 74.0 | 75.7 |
| Ceftriaxone | 70.7 | 49.5 | 47.9 | 52.1 | 54.2 | 50.3 | 52.8 | 60.6 | 60.9 | 61.0 | 57.0 | 62.7 |
| Levofloxacin | 94.8 | 72.2 | 74.3 | 77.6 | 78.9 | 79.3 | 76.8 | 75.5 | 79.7 | 86.2 | 78.0 | 83.6 |
| Meropenem | [3] (3) | [4] (4) | 93.2 (191) | 96.7 (483) | 93.1 | 92.3 | 99.2 | 100 | 94.2 | 93.5 | 99.0 | 96.2 |
| Minocycline | 89.7 | 75.8 | 72.5 | 65.6 | 57.4 | 58.1 | 50.2 | 28.7 | 75.4 | 74.8 | 74.0 | 83.6 |
| Pip-taz | 84.5 | 66.2 | 65.5 | 71.7 | 69.8 | 70.7 | 74.6 | 79.8 | 76.8 | 76.4 | 69.0 | 81.5 |
| Tigecycline | 98.3 | 95.5 | 96.1 | 96.9 | 95.9 | 95.3 | 94.4 | 98.9 | 92.8 | 95.1 | 98.0 | 93.8 |
| ***S. marcescens*** | **N = 25** | **N = 101** | **N = 179** | **N = 187** | **N = 312** | **N = 311** | **N = 190** | **N = 25** | **N = 31** | **N = 56** | **N = 43** | **N = 117** |
| Amikacin | 88.0 | 77.2 | 83.8 | 81.3 | 81.4 | 82.0 | 83.2 | 96.0 | 83.9 | 94.6 | 90.7 | 88.9 |
| Amox-clav | 8.0 | 1.0 | 6.7 | 9.1 | 4.2 | 3.2 | 6.3 | 0.0 | 3.2 | 3.6 | 7.0 | 3.4 |
| Ampicillin | 0.0 | 1.0 (100) | 4.5 (178) | 5.3 | 2.6 | 1.6 | 1.6 | 0.0 | 3.2 | 10.7 | 0.0 | 1.7 |
| Cefepime | 84.0 | 73.3 | 77.7 | 76.5 | 75.3 | 73.6 | 73.7 | 80.0 | 64.5 | 83.9 | 74.4 | 83.8 |
| Ceftriaxone | 80.0 | 55.4 | 67.0 | 69.5 | 68.6 | 66.2 | 58.9 | 72.0 | 54.8 | 76.8 | 72.1 | 77.8 |
| Levofloxacin | 96.0 | 82.2 | 84.9 | 87.7 | 87.2 | 82.3 | 78.9 | 84.0 | 87.1 | 89.3 | 74.4 | 92.3 |
| Meropenem | [1] (1) | [6] (6) | 91.7 (72) | 95.1 (183) | 95.2 | 95.2 | 93.7 | 100 | 96.8 | 96.4 | 95.3 | 95.7 |
| Minocycline | 100 | 86.1 | 88.8 | 62.0 | 50.3 | 50.2 | 42.6 | 52.0 | 93.5 | 92.9 | 69.8 | 90.6 |
| Pip-taz | 92.0 | 81.2 | 79.3 | 83.4 | 83.7 | 84.2 | 76.3 | 88.0 | 96.8 | 94.6 | 83.7 | 88.9 |
| Tigecycline | 100 | 97.0 | 97.2 | 97.3 | 93.9 | 94.5 | 91.1 | 100 | 93.5 | 94.6 | 88.4 | 94.9 |
| ***P. aeruginosa*** | **N = 59** | **N = 169** | **N = 427** | **N = 384** | **N = 732** | **N = 753** | **N = 479** | **N = 66** | **N = 66** | **N = 117** | **N = 89** | **N = 272** |
| Amikacin | 76.3 | 68.6 | 75.6 | 66.4 | 71.4 | 70.8 | 71.6 | 59.1 | 71.2 | 83.8 | 92.1 | 83.8 |
| Amox-clav | - | - | - | - | - | - | - | - | - | - | - | - |
| Ampicillin | - | - | - | - | - | - | - | - | - | - | - | - |
| Cefepime | 64.4 | 55.6 | 63.9 | 53.9 | 59.3 | 59.0 | 58.2 | 45.5 | 74.2 | 65.8 | 75.3 | 69.5 |
| Ceftazidime | 64.4 | 56.2 | 63.0 | 47.1 | 54.2 | 54.8 | 49.3 | 47.0 | 63.6 | 68.4 | 84.3 | 71.3 |
| Ceftriaxone | - | - | - | - | - | - | - | - | - | - | - | - |
| Levofloxacin | 44.1 | 47.9 | 52.2 | 49.5 | 53.1 | 54.4 | 50.7 | 47.0 | 68.2 | 67.5 | 59.6 | 59.6 |
| Meropenem | [0] (3) | [3] (3) | 46.0 (187) | 51.8 | 55.6 | 53.0 | 53.2 | 43.9 | 51.5 | 60.7 | 50.6 | 61.4 |
| Minocycline | - | - | - | - | - | - | - | - | - | - | - | - |
| Pip-taz | 66.1 | 55.0 | 63.5 | 54.4 | 54.8 | 58.0 | 49.5 | 45.5 | 71.2 | 69.2 | 79.8 | 73.2 |
| Tigecycline | - | - | - | - | - | - | - | - | - | - | - | - |
| ***P. aeruginosa,* MDR** | **N = 17** | **N = 40** | **N = 94** | **N = 124** | **N = 200** | **N = 206** | **N = 161** | **N = 29** | **N = 13** | **N = 22** | **N = 7** | **N = 53** |
| Amikacin | 35.3 | 22.5 | 25.5 | 17.7 | 25.5 | 18.0 | 37.9 | 10.3 | 7.7 | 18.2 | [3] | 30.2 |
| Amox-clav | - | - | - | - | - | - | - | - | - | - | - | - |
| Ampicillin | - | - | - | - | - | - | - | - | - | - | - | - |
| Cefepime | 0.0 | 15.0 | 5.3 | 4.8 | 9.0 | 7.8 | 15.5 | 13.8 | 15.4 | 4.5 | [0] | 3.8 |
| Ceftazidime | 5.9 | 15.0 | 4.3 | 8.1 | 11.0 | 7.8 | 6.8 | 6.9 | 15.4 | 9.1 | [1] | 13.2 |
| Ceftriaxone | - | - | - | - | - | - | - | - | - | - | - | - |
| Levofloxacin | 0.0 | 0.0 | 2.1 | 0.8 | 1.0 | 1.9 | 5.0 | 0.0 | 23.1 | 0.0 | [1] | 0.0 |
| Meropenem | [0] (3) | –– | 7.0 (43) | 4.8 | 3.0 | 2.9 | 7.5 | 10.3 | 7.7 | 9.1 | [0] | 1.9 |
| Minocycline | - | - | - | - | - | - | - | - | - | - | - | - |
| Pip-taz | 5.9 | 10.0 | 12.8 | 12.9 | 11.0 | 13.6 | 6.2 | 6.9 | 15.4 | 9.1 | [1] | 15.1 |
| Tigecycline | - | - | - | - | - | - | - | - | - | - | - | - |
| ***A. baumannii*** | **N = 54** | **N = 135** | **N = 246** | **N = 284** | **N = 470** | **N = 487** | **N = 312** | **N = 43** | **N = 45** | **N = 77** | **N = 60** | **N = 141** |
| Amikacin | 11.1 | 29.6 | 40.2 | 26.1 | 29.1 | 29.4 | 29.2 | 18.6 | 31.1 | 29.9 | 31.7 | 50.4 |
| Amox-clav | - | - | - | - | - | - | - | - | - | - | - | - |
| Ampicillin | - | - | - | - | - | - | - | - | - | - | - | - |
| Cefepime | 7.4 | 21.5 | 29.3 | 21.1 | 28.1 | 26.3 | 16.0 | 7.0 | 6.7 | 11.7 | 3.3 | 18.4 |
| Ceftazidime | 3.7 | 15.6 | 22.8 | 13.7 | 18.9 | 18.9 | 14.4 | 14.0 | 8.9 | 11.7 | 15.0 | 23.4 |
| Ceftriaxone | 0.0 | 3.7 | 11.4 | 8.8 | 14.0 | 10.5 | 5.4 | 2.3 | 4.4 | 2.6 | 1.7 | 11.3 |
| Levofloxacin | 5.6 | 14.1 | 24.4 | 18.3 | 23.6 | 22.0 | 15.1 | 11.6 | 6.7 | 9.1 | 18.3 | 20.6 |
| Meropenem | [0] (1) | [0] (2) | 34.7 (124) | 29.6 | 30.6 | 28.5 | 20.2 | 11.6 | 11.1 | 10.4 | 10.0 | 20.6 |
| Minocycline | 98.1 | 100 | 92.7 | 94.7 | 83.6 | 89.1 | 79.5 | 88.4 | 86.7 | 89.6 | 93.3 | 83.0 |
| Pip-taz | 3.7 | 17.0 | 22.0 | 18.0 | 21.1 | 17.9 | 12.2 | 4.7 | 2.2 | 10.4 | 13.3 | 22.7 |
| Tigecycline | - | - | - | - | - | - | - | - | - | - | - | - |
| ***A. baumannii,* MDR** | **N = 40** | **N = 66** | **N = 131** | **N = 209** | **N = 343** | **N = 344** | **N = 240** | **N = 38** | **N = 39** | **N = 62** | **N = 48** | **N = 94** |
| Amikacin | 2.5 | 4.5 | 9.9 | 6.2 | 7.0 | 11.0 | 16.3 | 10.5 | 20.5 | 19.4 | 18.8 | 28.7 |
| Amox-clav | - | - | - | - | - | - | - | - | - | - | - | - |
| Ampicillin | - | - | - | - | - | - | - | - | - | - | - | - |
| Cefepime | 5.0 | 1.5 | 5.3 | 1.4 | 9.9 | 9.6 | 2.5 | 0.0 | 0.0 | 1.6 | 2.1 | 0.0 |
| Ceftazidime | 2.5 | 0.0 | 3.1 | 3.8 | 3.5 | 7.3 | 5.4 | 2.6 | 5.1 | 0.0 | 4.2 | 3.2 |
| Ceftriaxone | 0.0 | 1.5 | 0.0 | 0.0 | 0.9 | 0.6 | 0.0 | 0.0 | 0.0 | 0.0 | 0.0 | 0.0 |
| Levofloxacin | 0.0 | 0.0 | 1.5 | 2.9 | 1.2 | 2.3 | 2.1 | 2.6 | 0.0 | 0.0 | 0.0 | 0.0 |
| Meropenem | [0] (1) | [0] (2) | 8.2 (73) | 8.1 | 9.9 | 9.0 | 4.2 | 7.9 | 2.6 | 0.0 | 0.0 | 0.0 |
| Minocycline | 97.5 | 100 | 91.6 | 95.7 | 81.0 | 87.5 | 76.7 | 89.5 | 84.6 | 87.1 | 91.7 | 77.7 |
| Pip-taz | 0.0 | 6.1 | 1.5 | 1.4 | 2.0 | 2.0 | 1.7 | 0.0 | 0.0 | 0.0 | 0.0 | 1.1 |
| Tigecycline | - | - | - | - | - | - | - | - | - | - | - | - |
| ***H. influenzae*** | **N = 32** | **N = 127** | **N = 134** | **N = 276** | **N = 211** | **N = 210** | **N = 91** | **N = 71** | **N = 12** | **N = 42** | **N = 42** | **N = 52** |
| Amikacin | - | - | - | - | - | - | - | - | - | - | - | - |
| Amox-clav | 100 | 99.2 | 99.3 | 99.6 | 98.1 | 100 | 98.9 | 100 | 100 | 100 | 100 | 100 |
| Ampicillin | 75.0 | 85.8 | 76.1 | 76.4 | 77.3 | 80.5 | 74.7 | 71.8 | 83.3 | 81.0 | 69.0 | 80.8 |
| Cefepime | 100 | 98.4 | 99.3 | 99.3 | 100 | 100 | 97.8 | 100 | 100 | 100 | 95.2 | 100 |
| Ceftriaxone | 100 | 99.2 | 98.5 | 100 | 100 | 100 | 100 | 100 | 100 | 97.6 | 100 | 100 |
| Levofloxacin | 100 | 100 | 100 | 100 | 100 | 100 | 100 | 100 | 100 | 100 | 100 | 100 |
| Meropenem | –– | 100 (10) | 100 (61) | 100 (273) | 100 | 100 | 100 | 100 | 100 | 100 | 100 | 100 |
| Minocycline | 100 | 97.6 | 100 | 99.3 (275) | 97.6 | 97.6 | 98.9 | 100 | 100 | 100 | 100 | 100 |
| Pip-taz | 100 | 99.2 | 100 | 99.3 | 99.5 | 98.1 | 100 | 98.6 | 100 | 100 | 100 | 100 |
| Tigecycline | 100 | 100 | 97.8 | 96.0 | 99.1 | 97.6 | 100 | 90.1 | 100 | 97.6 | 100 | 100 |
| ***H. influenzae*, βLPos** | **N = 8** | **N = 18** | **N = 29** | **N = 61** | **N = 46** | **N = 34** | **N = 23** | **N = 19** | **N = 1** | **N = 8** | **N = 13** | **N = 10** |
| Amikacin | - | - | - | - | - | - | - | - | - | - | - | - |
| Amox-clav | [8] | 94.4 | 96.6 | 100 | 97.8 | 100 | 95.7 | 100 | [1] | [8] | 100 | 100 |
| Ampicillin | [0] | 0.0 | 0.0 | 1.6 | 0.0 | 2.9 | 0.0 | 0.0 | [0] | [0] | 0.0 | 0.0 |
| Cefepime | [8] | 94.4 | 100 | 98.4 | 100 | 100 | 95.7 | 100 | [1] | [8] | 100 | 100 |
| Ceftriaxone | [8] | 94.4 | 100 | 100 | 100 | 100 | 100 | 100 | [1] | [8] | 100 | 100 |
| Levofloxacin | [8] | 100 | 100 | 100 | 100 | 100 | 100 | 100 | [1] | [8] | 100 | 100 |
| Meropenem | –– | [2] (2) | 100 (21) | 100 (59) | 100 | 100 | 100 | 100 | [1] | [8] | 100 | 100 |
| Minocycline | [8] | 88.9 | 100 | 98.4 | 100 | 97.1 | 95.7 | 100 | [1] | [8] | 100 | 100 |
| Pip-taz | [8] | 94.4 | 100 | 100 | 100 | 100 | 100 | 100 | [1] | [8] | 100 | 100 |
| Tigecycline | [8] | 100 | 100 | 98.4 | 97.8 | 94.1 | 100 | 100 | [1] | [8] | 100 | 100 |

‘––‘ No isolates were tested against this agent.

‘-‘ no CLSI breakpoints available.

When N<10 percentages are not given, instead the total number of susceptible isolates are shown in square brackets.

When fewer than the total number of isolates collected were tested against a given antimicrobial, the number of isolates tested are shown in parentheses after the percentage susceptibility value.

Amox-clav, amoxicillin-clavulanic acid; βLPos, β-lactamase positive; ESBL, extended-spectrum β-lactamase; MDR, multidrug-resistant; pip-taz, piperacillin-tazobactam.
